# Heterologous Immunity Between SARS-CoV-2 and Pathogenic Bacteria

**DOI:** 10.3389/fimmu.2022.821595

**Published:** 2022-01-27

**Authors:** Peter J. Eggenhuizen, Boaz H. Ng, Janet Chang, Rachel M.Y. Cheong, Anusha Yellapragada, Wey Y. Wong, Yi Tian Ting, Julie A. Monk, Poh-Yi Gan, Stephen R. Holdsworth, Joshua D. Ooi

**Affiliations:** ^1^ Centre for Inflammatory Diseases, Department of Medicine, Monash Medical Centre, School of Clinical Sciences, Monash University, Clayton, VIC, Australia; ^2^ Department of Immunology, Monash Health, Monash Medical Centre, Clayton, VIC, Australia

**Keywords:** COVID-19, pathogenic bacteria, heterologous immunity, SARS-CoV-2 vaccine, cross-reactivity, memory T cell

## Abstract

Heterologous immunity, when the memory T cell response elicited by one pathogen recognizes another pathogen, has been offered as a contributing factor for the high variability in coronavirus disease 2019 (COVID-19) severity outcomes. Here we demonstrate that sensitization with bacterial peptides can induce heterologous immunity to severe acute respiratory syndrome coronavirus 2 (SARS-CoV-2) derived peptides and that vaccination with the SARS-CoV-2 spike protein can induce heterologous immunity to bacterial peptides. Using *in silico* prediction methods, we identified 6 bacterial peptides with sequence homology to either the spike protein or non-structural protein 3 (NSP3) of SARS-CoV-2. Notwithstanding the effects of bystander activation, *in vitro* co-cultures showed that all individuals tested (*n*=18) developed heterologous immunity to SARS-CoV-2 peptides when sensitized with the identified bacterial peptides. T cell recall responses measured included cytokine production (IFN-γ, TNF, IL-2), activation (CD69) and proliferation (CellTrace). As an extension of the principle of heterologous immunity between bacterial pathogens and COVID-19, we tracked donor responses before and after SARS-CoV-2 vaccination and measured the cross-reactive T cell responses to bacterial peptides with similar sequence homology to the spike protein. We found that SARS-CoV-2 vaccination could induce heterologous immunity to bacterial peptides. These findings provide a mechanism for heterologous T cell immunity between common bacterial pathogens and SARS-CoV-2, which may explain the high variance in COVID-19 outcomes from asymptomatic to severe. We also demonstrate proof-of-concept that SARS-CoV-2 vaccination can induce heterologous immunity to pathogenic bacteria derived peptides.

## Introduction

Heterologous immunity is when a memory T cell response elicited by one pathogen recognizes another pathogen. Heterologous immunity arising from epitope homology between severe acute respiratory syndrome coronavirus 2 (SARS-CoV-2) and other human coronaviruses has been identified as a mode of cross-protection from coronavirus disease 2019 (COVID-19) (1) (2). Additionally, individuals unexposed to SARS-CoV-2 have been shown to possess SARS-CoV-2-specific T cells (3–8). Heterologous immunity between other bacteria, such as *Mycobacterium bovis*, the bacteria contained in Bacille Calmette-Guérin vaccine (BCG), and SARS-CoV-2 has been shown as a mechanism of heterologous T cell cross-reactive immunity (9). Therefore, heterologous immunity is offered as one explanation for the variable outcomes of COVID-19 severity. However, the mechanism of heterologous immunity between SARS-CoV-2 and pathogenic bacteria has not been characterised.

Common bacterial pathogens encountered by humans throughout life produce symptoms ranging from mild to severe depending on the bacterium involved, the site of infection and the ability of the individual to mount a successful immune response to the infection. T cell involvement in clearing bacterial pathogens seeds memory T cells, which exhibit heightened functional responses upon re-infection yet can wane over time (10). These antigen-specific T cells may be able to cross-react with unrelated pathogens sharing regions of amino acid sequence homology that can act as a mimic in T cell recognition of peptide- major histocompatibility complex (MHC) in a phenomenon termed heterologous immunity (1). Re-infection with a different pathogen sharing homology regions therefore has the potential to activate these T cells and provide a level of heterologous immunity (11).

T cells forming part of an orchestrated immune response are crucial to combatting COVID-19 as COVID-19 convalescent individuals have been shown to exhibit SARS-CoV-2 specific T cell memory, whereas T cell imbalance and dysfunction is linked to severe manifestations of COVID-19 (12, 13).

In this study, we assessed *via* an *in vitro* mechanism whether pathogenic bacterial-peptide-primed T cells exhibit cross-reactivity with SARS-CoV-2-peptide or protein homologues. We extended this mechanism using direct *ex-vivo* human blood samples to assess whether SARS-CoV-2 vaccination seeds memory T cells with the capacity to cross-react with these bacterial epitopes.

## Materials and Methods

### Blood Samples

Whole blood was collected from healthy donors unexposed to SARS-CoV-2 (n = 18; age: 21 to 42 years; sex: 50% [9/18] male, 50% female [9/18]). Donors were confirmed to be unexposed to SARS-CoV-2, i.e. did not display symptoms of COVID infection, did not have a prior COVID-19 infection, and were seronegative for IgG/M SARS-CoV-2 by SARS-CoV-2 Colloidal Gold Immunochromatography Assay (MyBioSource). Six of the 18 healthy donors were subsequently vaccinated either with AstraZeneca ChAdOx1-S (n = 1) or COMIRNATY BNT162b2 (mRNA) vaccine (n = 5). SARS-CoV-2 spike seroconversion was verified by IgG/M SARS-CoV-2 Colloidal Gold Immunochromatography Assay in all 6 donors and whole blood was collected for pre- and post-vaccination analyses.

### Ethics

The project was performed according to the Declaration of Helsinki and was approved by Monash University Human Research Ethics Committee (project ID 25834). All donors provided written informed consent.

### Immune Cell Isolation

Human peripheral blood mononuclear cells (PBMCs) were isolated from fresh whole blood by Lymphoprep density gradient medium in SepMate tubes (Stemcell) and cell number was enumerated by 0.4% (v/v) trypan blue (Sigma) on a haemocytometer. PBMCs were used for CD14+ CD16- monocyte isolation using EasySep Human Monocyte Isolation Kit (Stemcell). Freshly isolated monocytes were then differentiated into mature or immature dendritic cells (DCs) using ImmunoCult DC Culture Kit according to the manufacturer’s instructions (Stemcell). Thawed PBMCs were treated with 100μg/mL DNAse I solution (Stemcell) for 15 minutes at room temperature before downstream use. CD3+ T cells were isolated from PBMCs using EasySep Human T Cell Isolation Kit (Stemcell).

### Human Leukocyte Antigen Typing

High resolution (n=7) and low resolution (n=3) human leukocyte antigen (HLA) typing of class I and II alleles was performed by the Australian Red Cross Victorian Transplantation and Immunogenetics Service by next-generation sequencing (**Table S2**). To assess whether HLA typing was necessary, 8 donors were not HLA typed.

### Peptide Selection and Alignment

The SARS-CoV-2 proteome (sequence ID NC_045512.2) was interrogated for sequence homology to bacteria (taxid:2) using the National Center for Biotechnology Information (NCBI) Protein BLAST search (https://blast.ncbi.nlm.nih.gov/Blast.cgi). Results were filtered to include only bacteria reported to be pathogenic to humans based on a literature search and then filtering to include homology sequences of at least 15 amino acids in length, sufficiently long enough for recognition by T cells. The 6 homologous 15mers showing highest homology between SARS-CoV-2 and pathogenic bacteria were then selected for analysis. Specifically, protein sequences from SARS-CoV-2 non-structural protein 3 (NSP3, YP_009725299.1) and SARS-CoV-2 spike glycoprotein (YP_009724390.1) with homologous bacterial protein sequences to *K. pneumoniae* O-acetyl-ADP-ribose deacetylase (PLK93285.1), *E. coli* and *C. freundii* O-acetyl-ADP-ribose deacetylase (WP_016149912.1), *K. pneumoniae* serine acetyltransferase (STW66654.1), *S.* Enteritidis AraC family transcriptional regulator (EBV2373563.1), *E. faecalis* AAA family ATPase (WP_025192929.1), *S. aureus* AAA family ATPase (MVH71995.1), *K. grimontii* AAA family ATPase (WP_155004179.1), *C. difficile* putative phosphatase (VTR10613.1), *Clostridium* spp. macro domain containing protein (WP_039218766.1) and human CLIP (NP_001020330.1) were sourced from NCBI Protein Database (https://www.ncbi.nlm.nih.gov/protein/). SARS-CoV-2 and bacterial peptide homologues were aligned to reveal amino acid similarity and identity using EMBOSS Needle Pairwise Sequence Alignment using the BLOSUM62 matrix (14). EMBOSS Needle Pairwise Alignment was also used to align the bacterial 15mer sequences to known human coronaviruses using sequences from the NCBI Protein Database, namely SARS-CoV-2 ORF1ab (QHD43415.1), Severe Acute Respiratory Syndrome Coronavirus 1 (SARS-CoV) ORF1ab (NP_828849.7), Middle East Respiratory Syndrome Coronavirus (MERS) ORF1ab (YP_009047202.1), OC43 ORF1ab (YP_009555238.1), HKU1 ORF1ab (YP_173236.1), NL63 ORF1ab (YP_003766.2), 229E ORF1ab (NP_073549.1), SARS-CoV-2 spike (YP_009724390.1), SARS-CoV spike (YP_009825051.1), MERS spike (YP_009047204.1), OC43 spike (YP_009555241.1), HKU1 spike (YP_173238.1), NL63 spike (YP_003767.1) and 229E spike (NP_073551.1). SARS-CoV-2 variant analysis was performed on data obtained from Pangolin (https://cov-lineages.org/) and Centers of Disease Control and Prevention SARS-CoV-2 Variant Classifications and Definitions (https://www.cdc.gov/coronavirus/2019-ncov/variants/variant-info.html) using data available up to 1 January 2022.

### Peptide and Protein

**Figure 1:**
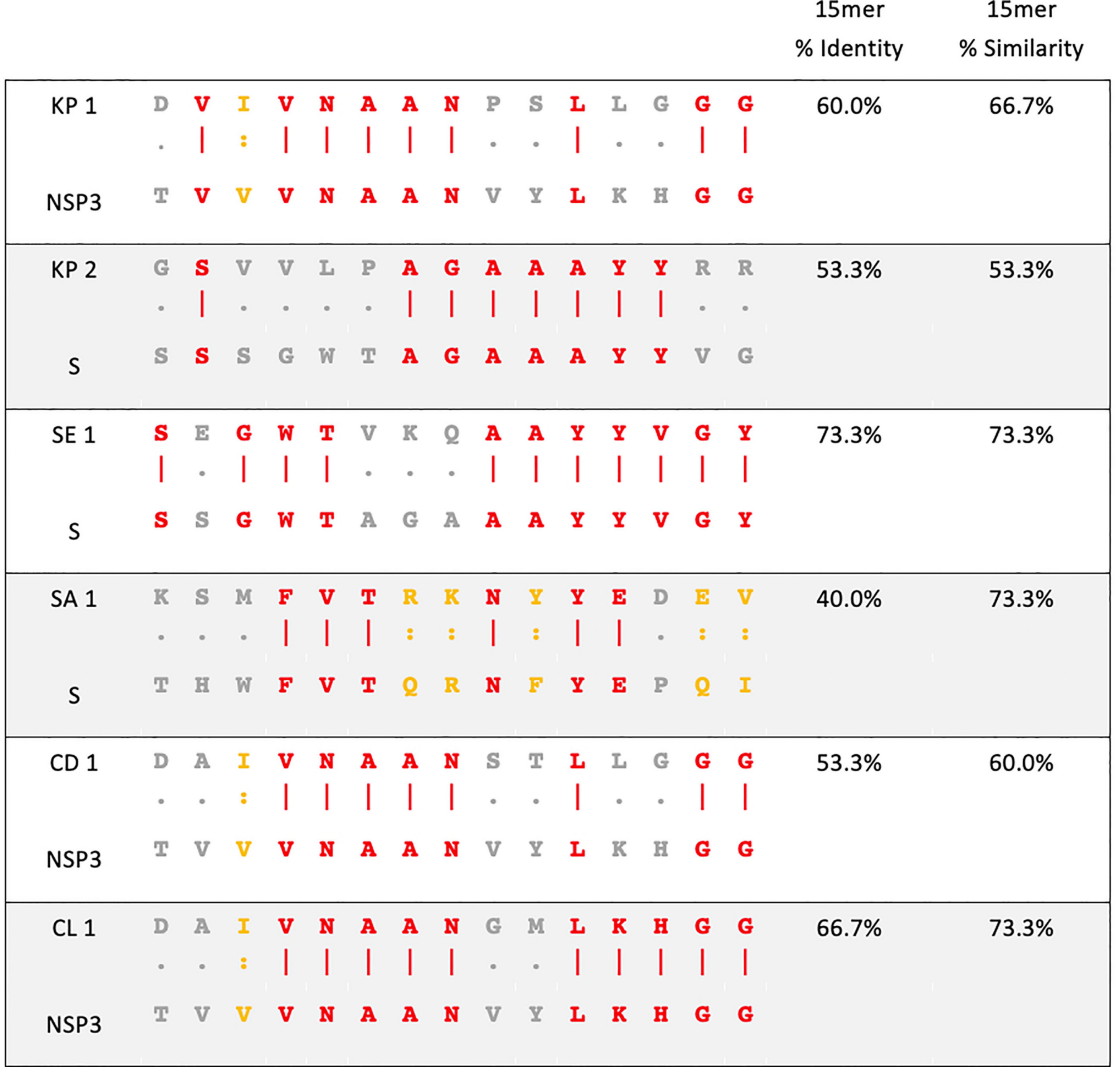
Sequence homology between pathogenic bacteria and SARS-CoV-2. Amino acid sequence alignment of pathogenic bacteria (top) and SARS-CoV-2 (bottom) used in this study. KP- *Klebsiella pneumoniae*, SE- *Salmonella* Enteritidis, SA- *Staphylococcus aureus*, CD- *Clostridioides difficile*, CL- *Clostridium* spp., NSP3- non-structural protein 3 of SARS-CoV-2, S- spike glycoprotein of SARS-CoV-2. Red amino acid- identity. Yellow amino acid- similarity. Grey amino acid- no identity or similarity.

Peptides were synthesised as 15mers with an N-terminus free amine (H-) and a free acid group at the C-terminus (-OH) (Mimotopes). Peptide purity was determined as ≥90% by reversed-phase high-performance liquid chromatography. Peptide sequences used in this study are listed in **Figure 1** and control peptide CLIP_103-117_ (PVSKMRMATPLLMQA). Peptides were reconstituted in 5% dimethyl sulfoxide (DMSO, Sigma) in sterile water (v/v). The final concentration of peptides used *in vitro* was 10μg/mL and 0.005% DMSO (v/v). SARS-CoV-2 recombinant proteins NSP3 and spike glycoprotein were purchased from MyBioSource. Proteins were >90% pure as determined by sodium dodecyl sulphate–polyacrylamide gel electrophoresis (SDS-PAGE) quantitative densitometry by Coomassie Blue staining. Proteins were reconstituted in sterile H_2_O and used *in vitro* at 10μg/mL.

### Peptide-HLA Binding Affinity

HLA allele global coverage of HLA-typed donors was calculated using Immune Epitope Database Analysis Resource – Population Coverage (15). NetMHCpan 4.1 was used across each bacterial homology region broken into 9mers overlapping by 1 amino acid for peptide- major histocompatibility complex (MHC) class I binding affinity. NetMHCIIpan 4.0 was used across each bacterial homology region broken into 15mers overlapping by 1 amino acid for peptide-MHC class II binding affinity (16). HLA alleles were chosen to reflect a globally representative collection of alleles as well as including the alleles of HLA-typed donors used in this study (17, 18).

### Intracellular Cytokine Staining Co-Culture

Intracellular cytokine staining (ICS) *in vitro* co-culture was established for assessing T cell cross reactivity, as reported previously (19, 20). Briefly, a completely autologous co-culture of 10^5^ freshly isolated human CD3+ T cells, 10^4^ human immature DCs pulsed with 10μg/mL of bacterial peptide (**Figure 1**) or control peptide (PVSKMRMATPLLMQA) was established in 96 well round bottom plates (100uL/well). Positive assay control received anti-human CD2, anti-human CD3, and anti-human CD28 coated MACS iBeads (Miltenyi). Negative assay control received no peptide. Five days later, the co-culture was supplemented with 40IU/mL recombinant human IL-2 (Stemcell) and re-incubated for 2 more days before resting the culture by washing twice with PBS and re-incubation in media without peptide. After 2 days resting with no IL-2 and no peptide, 10^4^ immature DCs pulsed with one of the SARS-CoV-2-peptides, recombinant SARS-CoV-2 spike protein, NSP3 protein or control peptide (PVSKMRMATPLLMQA) and 1μg/mL anti-human CD28 (clone CD28.2, eBioscience) were added. 1X protein transport inhibitor cocktail (eBioscience) was added. Positive assay control received anti-human CD2, anti-human CD3, and anti-human CD28 coated MACS iBeads (Miltenyi). Negative assay control received no peptide. Culture was incubated for 6 hours after which cells were harvested for flow cytometry analysis by ICS.

HLA blocking to verify the cross-reactive T cell responses arise from T cell interaction with peptide-MHC. HLA blocking for CD4+ T cells was achieved by mouse anti-human HLA DR/DP/DQ antibody (clone TU39, eBioscience). In instances where HLA typed individuals were *in silico* predicted to bind to only one MHC class II allele (e.g. only HLA-DP, and not -DR or -DQ), we blocked with an antibody specific for only that HLA molecule (e.g. mouse anti-human HLA-DP, clone B7/21, Abcam). HLA blocking on CD8+ T cells was achieved by mouse anti-human HLA-A/B/C antibody (clone W6/32, eBioscience). Isotype controls used were mouse IgG2a kappa (clone eBM2a, eBioscience) and mouse IgG3 (clone B10, eBioscience). Samples received 10μg/mL of individual blocking antibody to the immature DCs for 1 hour at 37°C in a 5% CO_2_ incubator before pulsing with SARS-CoV-2 peptide followed by the ICS culture and staining protocol.

Direct *ex vivo* pre- and post-vaccinated ICS cultures were established as above ICS protocol with CD3+ T cells, immature DCs and bacterial peptide except the co-culture was incubated for only 2 hours after addition of peptide, after which protein transport inhibitor was added for 6 hours followed by ICS by flow cytometry.

### Intracellular Cytokine Staining Flow Cytometry

**Figure 2:**
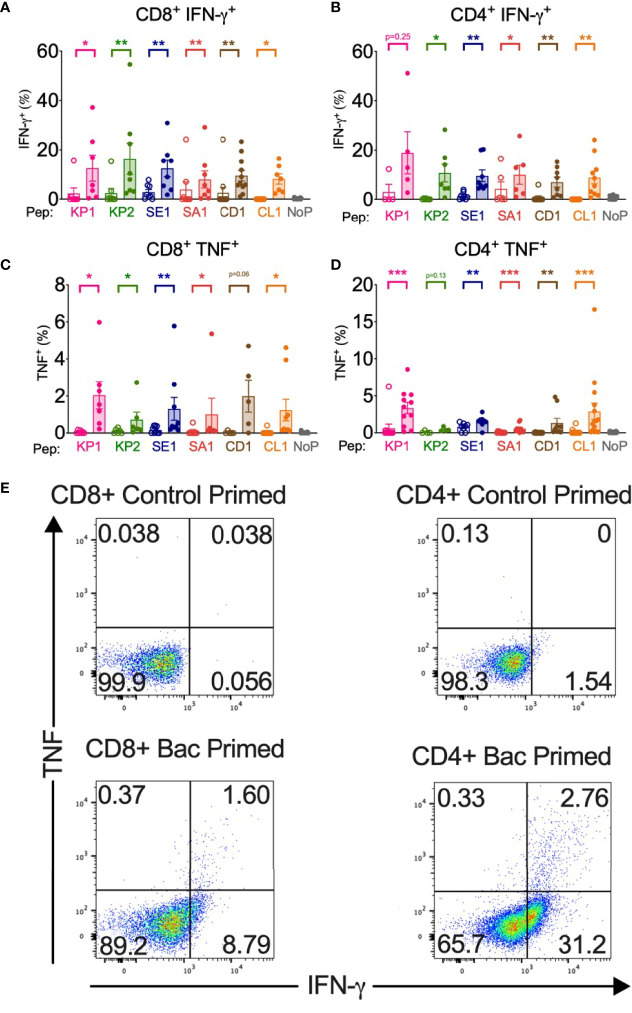
Bacterial peptide priming enhances SARS-CoV-2 T cell cytokine expression in responders. Pathogenic bacterial peptide **(bac)** primed T cells restimulated with SARS-CoV-2-peptide measured by flow cytometry with background control values subtracted. Unshaded bars- Control primed (irrelevant peptide, PVSKMRMATPLLMQA), then restimulated with SARS-CoV-2 homologous peptide. Shaded bars– bac primed (KP1, KP2, SE1, SA1, CD1 or CL1) then SARS-CoV-2-peptide homologue restimulated. NoP – no peptide negative control. **(A)** CD8+ IFN-γ+ responses (*n*=7-12), **(B)** CD4^+^IFN-γ^+^ responses (*n*=5-10), **(C)** CD8^+^TNF^+^ responses (*n*=5-9), **(D)** CD4^+^TNF^+^ responses (*n*=4-15), **(E)** Representative TNF (y-axis) and IFN-γ (x-axis) dot plots of a responder donor with their corresponding SARS-CoV-2 primary response control. *P < 0.05, **P < 0.01, ***P < 0.001 by Wilcoxon-matched-pairs-signed-rank test, comparing magnitude of response to SARS-CoV-2 peptides with or without bacterial peptide priming.

**Figure 3:**
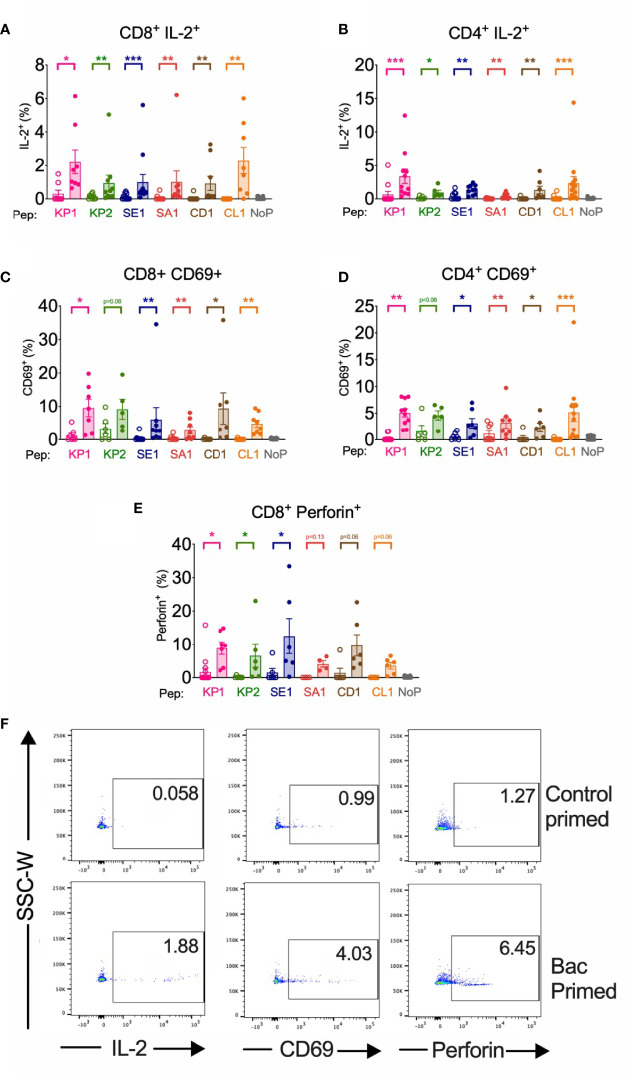
| Bacterial peptide priming enhances IL-2, CD69 and Perforin T cell responses against SARS-CoV-2 in responders. Pathogenic bacterial-peptide (KP1, KP2, SE1, SA1, CD1 or CL1) primed (shaded bars) or control primed with irrelevant peptide (unshaded bars) CD3^+^ T cells were restimulated with SARS-CoV-2-peptide-homologue-pulsed DCs for 6 hours and analysed by flow cytometry with background control values subtracted. NoP – no peptide negative control. *P < 0.05, **P < 0.01, ***P < 0.001 by Wilcoxon matched-pairs signed rank test. **(A)** CD8^+^ IL-2^+^ responses (n=8-12). **(B)** CD4^+^ IL-2^+^ responses (n=6-14). **(C)** CD8^+^ CD69^+^ responses (n=5-9). **(D)** CD4^+^ CD69^+^ responses (n=5-12). **(E)** CD8^+^ Perforin^+^ responses (n=4-15). **(F)** Representative donor T cell IL-2, CD69 and perforin dot plots of responder control-primed (top) and bacterial peptide (bac)-primed (bottom).

Cells were harvested and initially stained with Live/Dead Fixable Near Infra-Red Dead Cell Stain Kit (Invitrogen). Surface marker staining was performed with anti-human CD3 Brilliant Violet 510 (clone OKT3, Biolegend), anti-human CD4 allophycocyanin (APC, clone OKT4, eBioscience), anti-human CD8 Alexa Fluor 488 (clone HIT8a, Biolegend) and anti-human CD69 Brilliant UV 395 (clone FN50, BD) followed by fixation and permeabilisation using Transcription Factor Staining Buffer Set (eBioscience). Intracellular marker staining was performed with anti-human interferon-γ (IFN-γ) Phycoerythrin-Cyanine7 (PE Cy7, clone 4S.B3, eBioscience), anti-human tumour necrosis factor (TNF) Brilliant Violet 421 (clone Mab11, Biolegend), anti-human IL-2 Brilliant Blue 700 (clone MQ1-17H12, BD) and anti-human perforin Phycoerythrin (PE, clone B-D48, Biolegend). In some samples, to assess T memory phenotypes, anti-human CD45RA Brilliant Violet 786 (clone HI100, BD) was added and CD69-BUV395 was replaced with CCR7-BUV395 (clone 3D12, BD) and Perforin-PE was replaced with CD69-PE (clone FN50, BD). Then, CD45RA vs. CCR7 quadrant gating of Tcm, Tem, TEMRA, T naïve and T memory subsets was performed. Single color controls were prepared using UltraComp eBeads (Invitrogen) for single colour control antibodies and ArC amine reactive compensation bead kit (Invitrogen) for Live/Dead single colour control. After staining, cells were resuspended in PBS and acquired on an LSR-Fortessa X20 flow cytometer (BD) using BD FACSDiva software version 8.0.1. Samples were analysed in FlowJo 10.6.2. Fluorescence minus one (FMO) controls were used to determine positive gating and gating strategy is found in **Figure S3A**. Positive and negative controls indicated successful assay conditions. Positive control stimulation with anti-human CD2, anti-human CD3, and anti-human CD28 coated MACS iBeads indicated CD8+ T cells showed mean % positive of 70.85%, 7.89%, 4.24%, 51.99% and 61.2% for parameters IFN-γ+, TNF+ IL2+, CD69+ and perforin+, respectively. Positive control CD4+ T cells showed a mean % positive of 54.87%, 16.16%, 8.53% and 48.87% for parameters IFN-γ+, TNF+, IL-2+ and CD69+, respectively. Negative control stimulation with no peptide values are shown in **Figures 2** and **3**. Responder individuals showed positive staining after subtraction of the primary SARS-CoV-2 response control (control-peptide-primed, SARS-CoV-2-peptide restimulated). Non-responder individuals showed no positive staining after subtraction of the primary SARS-CoV-2 response control (**Figure S2**). For quantitative and statistical analysis (**Figures 2** and **3**), the responders were selected and had the restimulation background control (bacterial-peptide primed and irrelevant peptide restimulated) subtracted from the corresponding bacterial-peptide primed, SARS-CoV-2 test sample and the primary SARS-CoV-2 response control to remove any assay-related background responses.

### Proliferation Co-Culture

Proliferation co-culture was established using previously reported dual stain proliferation assay. Briefly, a completely autologous co-culture was set up with 10^5^ CD3+ T cells stained with Cell Trace Yellow (CTY, Invitrogen) and co-cultured with 10^4^ mature DCs pulsed with 10μg/mL bacterial peptide (**Figure 1**) or control peptide (PVSKMRMATPLLMQA) in 96 well round-bottom plate (Corning) at 100μL/well. After 7 days incubation at 37°C and 5% CO_2_, cells were rested by washing 2X with PBS and re-incubation in media without peptide for 2 days. 2 days later, the culture was stained with Cell Trace Violet (CTV, Invitrogen). Then 10^4^ SARS-CoV-2 peptide-pulsed mature DCs (or control pulsed) were added to the culture before incubation for 7 more days when they were harvested for flow cytometry analysis. At no stage was IL-2 added to the culture. Positive assay control received anti-human CD2, anti-human CD3, and anti-human CD28 coated MACS iBeads (Miltenyi) at the priming and restimulation steps. Negative assay control received no peptide at the priming and restimulation steps.

Pre- and post-vaccination proliferation assay was established as above with CD3+ T cells, mature DCs and bacterial peptides SA1, SE1 or KP2 (**Figure 1**). The T cells were stained initially with CTV only and cultured for 7 days after which CTVlo cells were analysed by flow cytometry and presented as raw data in Figure 6E.

### Proliferation Flow Cytometry

**Figure 4:**
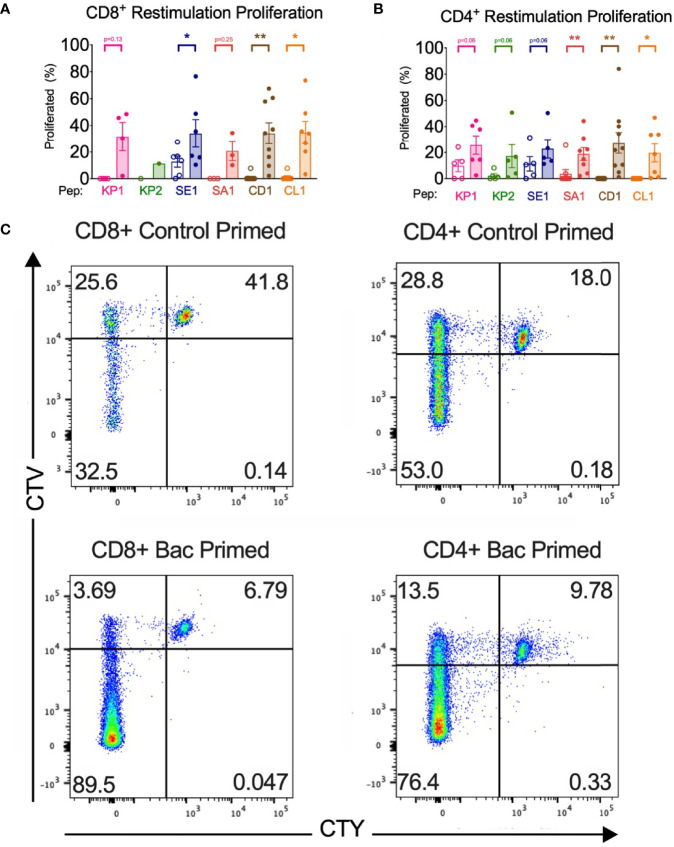
Bacterial peptide priming increases T cell proliferation in responders. **(A)** CD8+ proliferation from restimulation is enhanced by pathogenic bacterial peptide priming (*n*=1-10). **(B)** CD4+ proliferation from restimulation is enhanced by bacterial peptide priming (*n*=5-10). Unshaded bars- Control-primed (irrelevant peptide, PVSKMRMATPLLMQA), then SARS-CoV-2-peptide restimulated. Shaded bars- bacterial peptide primed then SARS-CoV-2-peptide homologue restimulated. *P < 0.05, **P < 0.01 by Wilcoxon-matched-pairs-signed rank test, comparing the magnitude of response to SARS-CoV-2 peptides with or without bacterial peptide priming after subtraction of the background control. **(C)** Representative CellTrace Yellow (CTY) vs. CellTrace Violet (CTV) dot plots of CD8+ and CD4+ cultured cells. Top right quadrant gate (CTY^hi^CTV^hi^ cells) did not proliferate upon priming or restimulation. Top left quadrant gate (CTY^lo^CTV^hi^ cells) proliferated upon priming but not with restimulation. Bottom left quadrant gate (CTY^lo^CTV^lo^ cells) proliferated upon both priming and restimulation.

Cells were harvested and initially stained with Live/Dead Fixable Near Infra-Red Dead Cell Stain Kit (Invitrogen). Surface marker staining was performed with anti-human CD3 PerCP (clone SK7, Biolegend), anti-human CD4 APC (clone OKT4, eBioscience), and anti-human CD8 Alexa Fluor 488 (clone HIT8a, Biolegend). Controls and gating and responder/non-responders were set up as for the ICS flow cytometry with the addition of Cell Trace Yellow and Violet single colour control using cultured cells. CTY vs. CTV gating was based on the point at which the first cell division took place visible by fluorescence dye dilution. CTY staining T cells before bacterial peptide stimulation, then staining with CTV pre SARS-CoV-2 peptide restimulation enabled us to determine if T cells primed with pathogenic bacterial peptides show enhanced T cell proliferation when restimulated with the SARS-CoV-2 homologous peptide by delineating the T cells which proliferated in priming only, in both priming and restimulation or not at all. Pre- and post-vaccinated proliferation assay was measured by CTV mean fluorescence intensity and presented as raw data (**Figure S6**). Gating strategy for proliferation assays is found in **Figure S3B**. Quantitative and statistical analysis was performed on the proportion of T cells in the CTY^lo^CTV^lo^ gate, being the T cells that underwent proliferation upon both priming and restimulation (**Figures 4A, B**). For such analysis, the responders were selected and had the restimulation background control (bacterial-peptide primed and irrelevant peptide restimulated) subtracted from the corresponding bacterial-peptide primed, SARS-CoV-2 test sample and the primary SARS-CoV-2 response control to remove any assay-related background stimulation.

### Activation-Induced Marker Assay

Activation-induced marker (AIM) assay was established according to previously published protocols (21, 22). Briefly, 500,000 PBMCs were seeded in 96 well U bottom plates (Corning) in 100uL RPMI 1640 (Gibco) supplemented with 10% autologous serum for 3 hours at 37°C, after which 0.5μg/mL of anti-human CD40 (clone HB14, Miltenyi) was added for 15 minutes at 37°C. 10μg/mL of bacterial peptide was then added and incubated for 20 hours at 37°C, after which cells were harvested for flow cytometry.

### Activation-Induced Marker Flow Cytometry

AIM markers for the detection of antigen-specific T cells were selected based on previously reported methods (21–23). Cells were harvested and initially stained with Live/Dead Fixable Aqua Dead Cell Stain Kit (Invitrogen). Surface marker staining was performed with anti-human CD3 PerCP (clone SK7, Biolegend), anti-human CD4 APC (clone OKT4, eBioscience), and anti-human CD8 Alexa Fluor 488 (clone HIT8a, Biolegend), anti-human CD69 PE (clone FN50, BD), anti-human CD45RA Brilliant Violet 711 (clone HI100, BD), anti-human CCR7 BUV395 (clone 3D12, BD), anti-human CD137 (4-1BB) Brilliant Violet 421 (clone 4B4-1, BD), anti-human CD134 (OX40) PE Cy7 (clone ACT-35, eBioscience) and anti-human CD154 (CD40L) APC Cy7 (clone TRAP1, BD). Control samples and sample acquisition were prepared as per ICS flow cytometry. AIM assay gating strategy was established (**Figure S3C**). Memory T cells were selected based on expression patterns of CD45RA and CCR7. Co-expression of CD45RA+ and CCR7+ allowed exclusion of the naïve T cell subset, leaving the T memory cells comprising subsets Tem, Tcm and TEMRA, for AIM analysis. On CD4+ T memory cells, AIM+ T cell responses were identified by co-expression of CD134 and CD137 or CD69 and CD154. On CD8+ T memory cells, AIM+ T cell responses were identified by co-expression of CD69 and CD137 or CD69 and CD154.

### Statistics

Flow cytometry data was processed in R Studio ver.1.3.959 and Graph Pad Prism 7 (Graphpad Software Inc.). A Shapiro-Wilk test to determine normality was performed before a two-tailed, Wilcoxon-matched-pairs-signed-rank test to compare responses of the bacterial primed T cell responders with control-primed responders and between pre- and post-vaccinated samples.

## Results

### SARS-CoV-2 Protein Homology With Pathogenic Bacteria

#### BLAST to Determine Regions of Homology

BLAST identified protein sequence homology regions between SARS-CoV-2 spike glycoprotein and NSP3 and pathogenic bacteria protein sequences. The bacterial sequences are present in common bacterial pathogens as well as commensal bacteria known to have the capacity to cause infections. Namely, these bacteria are *Klebsiella pneumoniae, K. grimontii, Escherichia coli, Salmonella* Enteritidis*, Enterococcus faecalis, Staphylococcus aureus, Citrobacter freundii, Clostridioides difficile*, formerly known as *Clostridium difficile, Clostridium haemolyticum* and *Clostridium novyi.*

#### Six Potential 15mer Peptide Pair Homologues Identified

The homology between pathogenic bacteria and SARS-CoV-2 15mer peptide sequences was 40% to 73.3% identity and 53.3% to 73.3% similarity (**Figure 1**). When the homology regions are processed as 9mers, a sufficient length for MHCI presentation to CD8+ T cells, they exhibited up to 100% similarity and 77.7% identity (**Table S1**).

#### Pathogenic Bacteria Homology With SARS-CoV-2 Variants of Concern and Other Coronaviruses

Pango lineage analysis of the current SARS-CoV-2 variants of concern B.1.1.7 (Alpha), B.1.351 (Beta), P.1 (Gamma), B.1.617.2 (Delta) and BA.1 (Omicron) and other variants of interest B.1.525 (Eta), B.1.526 (Iota), B.1.617.1 and A.23.1 showed no defining amino acid differences to the proteome reference sequence (ID NC_045512.2) at the epitopes overlapping with the regions of homology (**Table S4**). However, some sequences of variant B.1.617.2 (Delta variant) possess a S:W258L mutation (24). This affects the homology of KP2 and SE1 peptide pairs with the KP2 increasing in amino acid identity from 53.3% to 60% and SE1 decreasing in amino acid identity from 73.3% to 66.6%. Sequence alignment of pathogenic bacteria 15mers to other human coronaviruses like the more serious but less common SARS-CoV and MERS-CoV and the more common but less serious OC43, HKU1, NL63 and 229E revealed the 15mers shared some but never complete homology between the pathogenic bacteria 15mer, SARS-CoV-2 15mer and other coronaviruses (**Table S4**). Pathogenic bacteria sequences sharing homology with spike glycoprotein (KP2, SE1 and SA1) all shared more homology with SARS-CoV-2 than any of the other coronaviruses. Whereas pathogenic bacteria sequences sharing homology with NSP3 (KP1, CD1 and CL1) shared either more or less homology with other coronaviruses than SARS-CoV-2, depending on the coronavirus being aligned.

#### Bacterial Epitopes Have Broad MHCI and MHCII Binding Capacity

The *in silico* binding affinity of bacterial-derived epitopes to bind HLA alleles showed broad MHC class I and II binding capacity across a globally representative dataset. In addition, the HLA-typed donors represented a global MHC class I and II coverage of >97% and similar patterns of broad MHC class I and II binding affinity were observed (**Figures S1A, B**).

Based on both strong binding and high homology, 6 different 15mer peptide pairs from various bacteria were chosen for subsequent *in vitro* analysis (**Figure 1**).

### T cells Primed With Bacterial Peptides Have Enhanced Immune Responses to SARS-CoV-2-Peptide Homologues

Summary analysis of CD4+ and CD8+ T cells with the parameters IFN-γ, IL-2, TNF, perforin, CD69 and proliferation assay showed all individuals (n =18) exhibited a positive response in at least one parameter across all 6 tested peptide pairs when primed with bacterial peptide and restimulated with SARS-CoV-2 peptide homologue (**Figure S2**).

#### Increased Cytokine Responses in CD4+ and CD8+ T Cells

##### 15mer Homologues

CD8+ cytotoxic T cells showed significantly increased TNF, IFN-γ, and IL-2 production in at least 5 of the 6 peptide homologue pairs when pathogenic bacterial peptide primed compared with T cells primed with control peptide (**Figures 2**, **3**). Compared with control primed, TNF mean fold expression significantly increased in CD8+ T cells, except in CD1 (p= 0.06), ranging from 5.9-fold (KP2) to 65.6-fold (CD1) when bacterial peptide primed and SARS-CoV-2 restimulated. IFN-γ mean fold increase in expression from CD8+ T cells significantly increased in all peptide pairs and ranged from 4.1-fold (SA1) to 13.9-fold (KP2). CD8+ T cell IL-2 expression showed a significant mean fold increase from 5.7-fold (KP2) to 16.1-fold (CD1).

CD4+ helper T cells also showed a similar pattern of significantly increased TNF, IFN-γ and IL-2 expression in at least 5 of the 6 peptide homologue pairs (**Figures 2**, **3**). Responder individual CD4+ T cell responses showed IFN-γ significantly increased across 5 of the 6 peptide pairs, ranging from 5.7-fold (SA1) to 15.8-fold (KP1). 5 of the 6 peptide pairs showed a mean fold increase in TNF in CD4+ T cells ranging from 1-fold (SE1) to 132-fold (CD1). IL-2 expression in CD4+ T cells exhibited a significant mean fold increase in all 6 peptide pairs from 2.2-fold (SE1) to 17.9-fold (CL1).

##### Spike and NSP3 Protein

Recombinant NSP3 protein restimulation after bacterial peptide priming exhibited strong T cell cytotoxicity and thus no data could be generated for KP1, CD1 or CL1. Compared with control-primed and spike protein restimulated, bacterial peptide primed and spike glycoprotein restimulated T cells exhibited increased cytokine responses (**Figure S4**). TNF and IFN-γ responses were significantly increased with KP2 and SA1 primed CD4+ T cells but not SE1 primed CD4+ T cells (IFN-γ+ p = 0.06). IFN-γ responses were significantly increased with KP2 and SA1 primed CD8+ T cells whereas SE1 primed cells did not reach statistical significance (p=0.06). TNF responses were significant only with SE1 primed CD8+ T cells.

##### Blocking HLA Abolishes Cytokine Responses

As expected, blocking with isotype control antibody showed a positive T cell TNF and IFN-γ response, similar to no antibody treatment. HLA blocking of both MHCI- and MHCII-restricted T cell responses abolished this cytokine response indicating the cross-reactive T cell responses arise from peptide-MHC binding to the T cell receptor (TCR) on the T cell (**Figure S5**).

#### Potential Anti-Viral Response in CD8+ Cells

We found that in CD8+ T cells primed with pathogenic bacterial-derived peptides KP1, KP2 and SE1 had a significantly increased perforin expression upon SARS-CoV-2 restimulation when compared with control primed cells, whereas SA1, CD1 and CL1 failed to reach significance (p=0.1250, 0.06 and 0.06, respectively) (**Figures 3E, F**). The cross-reactive expression of perforin in responder individuals showed a fold-increase ranging from 4.3-fold (KP1) to 39.3-fold (KP2).

#### Early Activation in CD4+ and CD8+ T Cells

We found that compared with a SARS-CoV-2 primary immune response, the bacterial peptide primed T cells increased early activation marker CD69 surface expression across 5 of the 6 peptide pairs in both CD4+ and CD8+ T cells (**Figure 3**). CD69 expression exhibited a mean fold increase ranging from 1.8-fold (KP2) to 112-fold (CD1) in CD8+ cytotoxic T cells and from 1.8-fold (SA1) to 97.4-fold (CL1) in CD4+ T helper cells.

#### Enhanced Proliferation in CD4+ and CD8+ T Cells

All donor samples primed with bacterial peptide then SARS-CoV-2 peptide restimulated developed enhanced T cell proliferation in 5 of the 6 peptide homologue pairs (**Figure S2**). The magnitude of the enhanced proliferative response was quantified in the responders and compared with the proliferative response when control primed and SARS-CoV-2 restimulated with the background control subtracted. In 3 of the 6 tested peptide homologue pairs and across CD4+ and CD8+ T cells, a significant increase in T cell proliferation was observed when bacterial peptide primed and SARS-CoV-2 restimulated compared with control peptide primed and SARS-CoV-2 restimulated (**Figure 4**). T cell proliferation was enhanced in CD4+ cells with a mean fold increase of between 1.02-fold (SE1) and 6.63-fold (KP2) and in CD8+ T cells between 1.67-fold (SE1) and 41.6-fold (CD1). To quantify the initial proportion of T cells proliferating in response to the primary bacterial peptide stimulation, the proportion of CTV^lo^ T cells showed a mean CD8^+^ T cell proliferation of 33.1% to 59.3% across the 6 bacterial peptides and 3.8% for no peptide. CD4^+^ T cells showed a mean cell proliferation of 71.9% to 83.5% across the six bacterial peptides and 9.7% for no peptide (**Figure S6**).

An equal number of males (n=9) and females (n=9) were analysed in this study and no significant sex-specific differences were found in the parameters measured.

### T Cells Exposed to SARS-CoV-2 Vaccination Have Enhanced Immune Responses to Bacterial-Peptide Homologues

#### Increased Cytokine Responses in CD4+ and CD8+ T Cells

**Figure 5:**
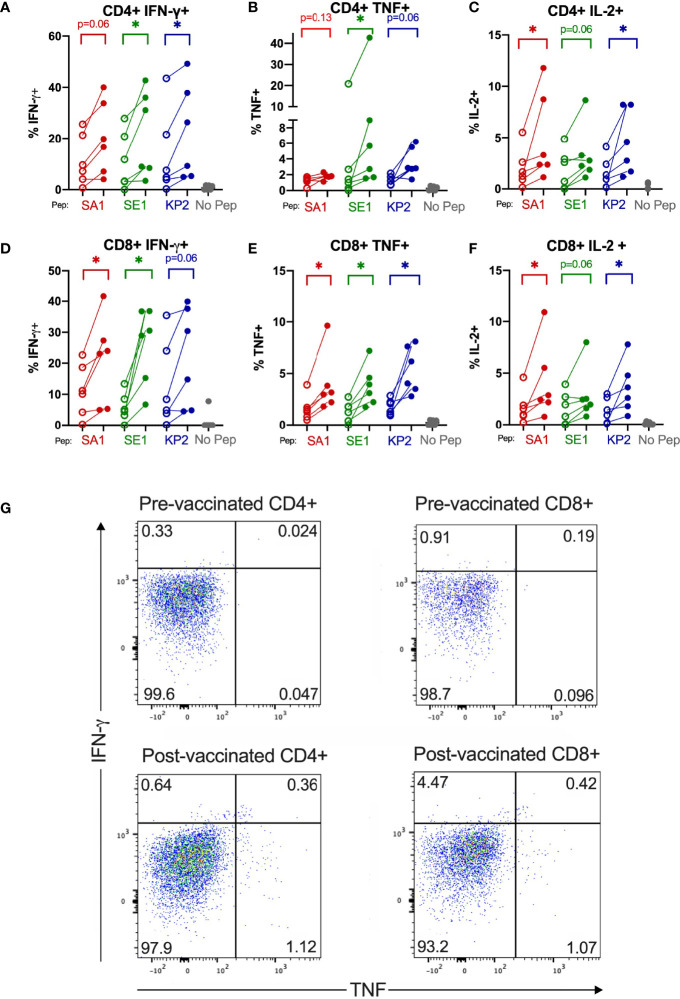
SARS-CoV-2 vaccination enhances T cell cytokine reactivity to pathogenic bacterial homologues. Direct *ex-vivo* T cell responses of donors (n=6) pre-SARS-CoV-2 vaccination (unshaded dots) and post-SARS-CoV-2 vaccination (shaded dots) to pathogenic bacterial peptides sharing homology with SARS-CoV-2 spike (SA1, SE1 and KP2) measured by intracellular cytokine staining. No Pep – no peptide negative control. *P < 0.05 by Wilcoxon matched-pairs signed rank test. **(A)** CD4^+^IFN-γ^+^ responses (n= 6). **(B)** CD4^+^TNF^+^ responses (n= 5-6). **(C)** CD4^+^IL-2^+^ responses (n= 6). **(D)** CD8^+^IFN-γ^+^ responses (n= 6). **(E)** CD8^+^TNF^+^ responses (n= 6). **(F)** CD8^+^IL-2^+^ responses (n= 6). **(G)** Representative donor T cell TNF (x-axis) and IFN-γ (y-axis) dot plots of pre-vaccinated CD4^+^ and CD8^+^ (top) and post-vaccinated CD4+ and CD8+ (bottom) responses to bacterial peptide SE1.

Direct *ex vivo* cytokine responses of bacterial-peptide-stimulated CD4+ and CD8+ T cells obtained before SARS-CoV-2 vaccination exhibited lower IFN-γ; TNF and IL-2 expression than T cells obtained from the same individuals (n=6) at least 2 weeks after their second SARS-CoV-2 vaccination (**Figure 5**). Compared with pre-SARS-CoV-2 vaccinated samples, IFN-γ+ expression increased by 46.8%, 77.6% and 41.9% in CD8+ cytotoxic T cells stimulated with SA1, SE1 and KP2 peptides, respectively, from post-SARS-CoV-2 vaccinated samples. CD8+ TNF+ expression increased by 58.8%, 65.1% and 67.3% for SA1, SE1 and KP2 peptide stimulation, respectively. Mean CD8+ IL-2+ expression increased by 58.7%, 45.6% and 61.2% for SA1-, SE1- and KP2-pulsed DC stimulation, respectively. On CD4+ T cells, IFN-γ+ showed a mean increase of 43.6%, 48.9% and 38.6% for peptides SA1, SE1 and KP2, respectively. Mean CD4+ TNF+ expression increased from pre- to post-vaccinated donors by 32.0%, 58.6% and 66.2% for SA1, SE1 and KP2, respectively. CD4+ IL-2+ expression increased by 59.4%, 43.8% and 65.8% for SA1, SE1 and KP2 pulsed DC stimulation, respectively. Overall, across the 18 parameters tested, 12 reached statistical significance and each of the 3 peptides showed statistical significance in 4 of the 6 parameters tested. Direct *ex vivo* cytokine responses of SARS-CoV-2-peptide stimulated T cells before and after SARS-CoV-2 vaccination showed post-SARS-CoV-2 vaccination responses were demonstrably higher than before vaccination (**Figure S8**).There was a significant difference between the before and after SARS-CoV-2 vaccination samples across the 3 tested SARS-CoV-2 peptide homologues in the parameters CD4+ IFN-γ+; CD4+ TNF+ and CD8+ TNF+ but not in CD8+ IFN-γ+ (p=0.06). Compared with the SARS-CoV-2-peptide-stimulated, post-vaccination samples, the bacterial-peptide-stimulated, post-vaccination samples exhibited similar yet slightly lower IFN-γ responses ranging from a 0.010 to 0.23 fold decrease in mean IFN-γ expression across both CD4+ and CD8+ T cells. TNF responses showed a greater decrease with bacterial peptide stimulated post-vaccination samples exhibiting a mean fold decease ranging from 0.20 to 0.61 across CD4+ and CD8+ T cells for the 3 peptide pairs tested.

#### IFN-γ+ or TNF+ T Cells Predominantly Memory T Cells

We found that most IFN-γ+ or TNF+ T cells isolated from post-vaccinated samples were of a memory phenotype, being T effector memory (Tem), T central memory (Tcm) or T effector memory re-expressing CD45RA (TEMRA) (**Figure S7**). Of the CD4+ IFN-γ+ *ex-vivo* T cells, 81.14%, 81.22% and 83.06% were of memory phenotype for SA1, SE1, KP2 stimulated, respectively. The CD69+ population of CD4+ cells compared with total CD4+ cells also exhibited an increase in memory cell populations Tem and Tcm and a decrease in naïve T cells.

#### Enhanced Proliferation in CD4+ and CD8+ T Cells

**Figure 6:**
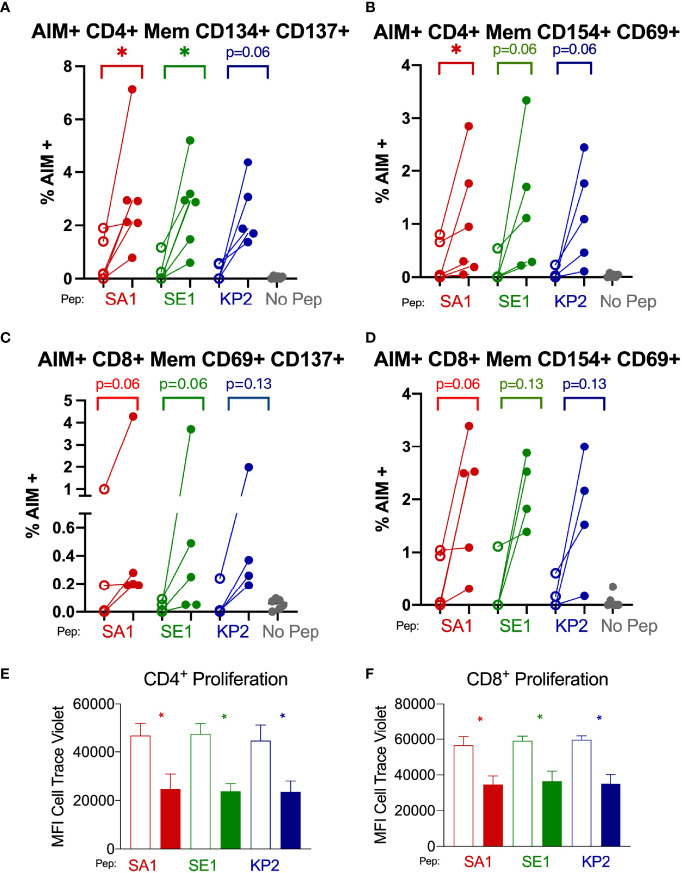
SARS-CoV-2 vaccination enhances T memory cell reactivity to pathogenic bacterial homologues by AIM and proliferation markers Direct *ex-vivo* T memory (Mem) cell responses of donors (n=6) pre-SARS-CoV-2 vaccination (unshaded dots) and post-SARS-CoV-2 vaccination (shaded dots) to pathogenic bacterial peptides sharing homology with SARS-CoV-2 spike (SA1, SE1 and KP2) measured by activation-induced markers (AIM) signifying antigen-specific responses **(A–D)**. 7-day proliferation assay of donors (n=6) pre-vaccination (unshaded bars) and post-vaccination (shaded bars) **(E, F)**. No Pep – no peptide negative control. MFI – mean fluorescence intensity. *P < 0.05 by Wilcoxon matched-pairs signed rank test. **(A)** AIM^+^ CD4^+^ T memory cell responses measured by co-expression of CD134 and CD137 (n= 5-6). **(B)** AIM^+^ CD4^+^ T memory responses measured by co-expression of CD154 and CD69 (n= 5-6). **(C)** AIM^+^ CD8^+^ T memory cell responses measured by co-expression of CD69 and CD137 (n= 4-5). **(D)** AIM^+^ CD8^+^ T memory cell responses measured by co-expression of CD154 and CD69 (n= 4-5). **(E)** CD4^+^ T cell proliferation in response to bacterial peptide homologues pre-and post SARS-CoV-2 vaccination (n= 6). **(F)** CD8^+^ T cell proliferation in response to bacterial peptide homologues pre-and post SARS-CoV-2 vaccination (n= 6).

We found that both CD4+ and CD8+ T cells underwent increased T cell proliferation in response to the 3 peptide homologues SA1, SE1 and KP2 in the post-vaccinated samples compared with the donor-matched pre-vaccinated sample as indicated by a dilution of proliferation dye as the cells undergo clonal expansion (**Figures 6E, F**). For CD4+ T cells, the mean fold decrease in CTV proliferation dye was -88.8%, -98.8% and -89.5% for SA1, SE1 and KP2, respectively. For CD8+ cytotoxic T cells, the mean decrease in CTV proliferation dye was -63.6%, -61.8% and -70.3% for SA1, SE1 and KP2, respectively. These results were significant in all 3 peptides for both CD4+ and CD8+ T cells.

#### Increased Antigen-Specific CD4+ and CD8+ T Memory Cells

After subtraction of the irrelevant peptide (PVSKMRMATPLLMQA) background stimulation control, we observed that when memory T cells were stimulated with the bacterial homologue peptides SA1, SE1 and KP2, the proportion of AIM+ cells increased in the post-SARS-CoV-2 vaccinated samples compared with pre-vaccinated (**Figures 6A–D**). AIM+ CD69+ CD154+ CD8+ memory T cells showed a mean increase of 79.4%, 87.1% and 88.9% for SA1, SE1 and KP2 peptide stimulation, respectively. Alternate AIM+ markers on CD8+ memory T cells by CD137+ CD69+ showed a mean increase of 76.7%, 96.7% and 91.1% for SA1, SE1 and KP2 peptide stimulation, respectively. AIM+ CD4+ memory T cells by CD134+ and CD137+ co-expression exhibited a mean increase of 79.6%, 91.3% and 90.9% % for SA1, SE1 and KP2 peptide stimulation, respectively. Alternate CD4+ AIM markers CD154+ and CD69+ on memory T cells exhibited a mean increase of 75.3%, 91.7% and 95.6% % for SA1, SE1 and KP2 peptide stimulation, respectively.

## Discussion

Heterologous immunity between SARS-CoV-2 and pathogenic bacteria may offer a mechanistic explanation for the diverse and unpredictable nature of COVID-19 disease severity. Six pathogenic bacteria derived epitopes sharing homology with SARS-CoV-2 spike or NSP3 protein have been shown to bind a broad set of HLA alleles and induce T cell cross reactivity in CD4+ and CD8+ T cells *in vitro*. Furthermore, *ex vivo* responses of memory T cells post-SARS-CoV-2 vaccination exhibited enhanced immune responses to bacterial-peptide homologues.

### SARS-CoV-2 Protein Homology With Pathogenic Bacteria

T cell-dependent heterologous immunity can arise *via* TCR-independent or TCR-dependent pathways. TCR-independent heterologous immunity results from non-specific, virus-induced activation of cytokines, such as IL-18 and IL-12, that occurs through bystander activation and without TCR involvement (25, 26). The cytokines produced during the second infection stimulate the memory T cells from the first infection. TCR-dependent heterologous immunity results from direct cross-reactivity between unrelated pathogens, whereby an initial infection or immunisation produces memory T cells that cross-react with antigens from a second, different infection (11). Memory T cells can persist for years yet wane over time meaning prior exposure from past infections may impact the response to current infections. The phenomenon of TCR-dependent heterologous immunity is the mechanism we explored for cross-reactive responses between pathogenic bacteria and SARS-CoV-2.

In this study, we identified 6 epitopes found in a variety of pathogenic bacteria that share significant homology with spike glycoprotein or NSP3 of SARS-CoV-2 (**Figure 1**). These epitopes were assessed as 15mers, which are of appropriate length to be presented by MHC class II to CD4+ T cells. The 15mers consist of seven 9mers overlapping by one amino acid (**Table S1**), which, through antigen presentation by dendritic cells (DCs), are able to be presented to CD8+ T cells by MHC class I.

The pathogenic bacteria that share homology with SARS-CoV-2 are *K. pneumoniae, K. grimontii, E. coli, S.* Enteritidis*, E. faecalis, S. aureus, C. freundii, C. difficile, C. haemolyticum* and *C. novyi.* They can cause infections ranging from common to rare and previous research has demonstrated T cell involvement in combatting the infections (27–46). They also have the potential to respond *via* heterologous mechanisms to cross-reactive epitopes and some of these bacteria have been isolated from the lower airways of COVID-19 patients (**Table S3**).

Recently, Sulaiman and colleagues studied the microbial signatures in the anatomically relevant lower airways of patients with severe COVID-19 requiring mechanical ventilation (47). Risk analysis from 589 respiratory cultures taken during hospitalization showed that bacterial culture positivity for *S. aureus, E.coli* and *K. pneumoniae* was associated with increased odds of survival in individuals who survived severe COVID-19 compared with those who died from COVID-19. This resulted in odds ratios of <1 suggesting severe COVID-19 patients who are positive for these bacteria are at less risk of death than those who do not carry the bacteria in their lung. The same study also analysed the microbiome of 142 bronchoalveolar lavage samples and found no statistically significant association between clinical outcomes and culture positivity, however there was a trend towards an increased rate of positive respiratory cultures for *S. aureus*, and *K. pneumoniae* in the survival groups. RNAseq analysis of the bronchoalveolar lavage samples showed a higher differential abundance of *C. difficile* in severe COVID-19 survivors compared with deceased, whereas *S. aureus* in such analysis remained unchanged. The presence of these pathogenic bacteria in severe COVID-19 survivors compared with those who died is interesting because typically bacterial co-infection in other viral respiratory pandemics, such as 1918 and 2009 H1N1 influenza, led to poor prognosis (48, 49). Furthermore, the increased bacterial burden and presence of gut-associated bacteria in the lung also typically leads to poor prognosis in acute respiratory distress syndrome (50, 51). Therefore, the cross-reactive T cell epitopes we have identified may offer a mechanistic explanation for heterogeneous outcomes of COVID-19 disease severity and mortality, particularly in the case of *S.aureus, E.coli, K.pneumoniae* and *C.difficile* co-infection.

The identified bacteria share epitopes with a significant degree of homology to NSP3 or spike glycoprotein of SARS-CoV-2 (**Figure 1**). NSP3 is a papain-like protease that regulates SARS-CoV-2 viral spread and shares homology with O-acetyl-ADP-ribose deacetylase_17-31_ from bacteria in the family Enterococcaceae, namely *K. pneumoniae, C. freundii* and *E.coli* (KP1) (52). NSP3 also shares homology with putative phosphatase_17-31_ of *C.difficile* (CD1) and macro domain-containing protein_22-36_ of *Clostridium* spp. such as *C.novyi* and *C.haemolyticum* (CL1). A recent study showed the NSP3 protein was the 4^th^ most immunodominant SARS-CoV-2 protein for CD4+ T cells and the second most immumodominant protein for CD8+ T cells while spike protein was the most immunodominant SARS-CoV-2 protein for both CD4+ and CD8+ T cells (22).

The spike glycoprotein of SARS-CoV-2 is responsible for catalysing the fusion between viral and target cell membranes to initiate infection and is the key component of SARS-CoV-2 vaccines as well as a target of other therapeutics for COVID-19 (53). The spike protein shares homology with serine acetyltransferase_69-83_ from *K.pneumoniae* (KP2), AraC family transcriptional regulator_279-293_ from *S*.Enteritidis (SE1) and AAA family ATPase_80-94_ from multiple species of family Enterobacteriaceae such as *S.aureus, E.faecalis and K.grimontii* (SA1). These regions of homology overlap with already characterised immunodominant T cell epitopes of SARS-CoV-2 spike (S) protein, namely S_258-266_, S_261-269_ and S_1101-1114_ as MHC class I restricted and S_1101-1115_ as the class II restricted immunodominant epitope (22). The immunodominant nature of these epitopes highlights their significance in SARS-CoV-2 immunity and their potential role in T cell cross-reactivity with homologous pathogenic bacterial epitopes.

Cross reactivity between other human coronaviruses and SARS-CoV-2 has been characterized and offered as an explanation for the variance of responses to COVID-19 (2). Cross reactivity of the epitopes in this study and other coronaviruses may be relevant in the bacterial epitopes sharing homology with NSP3 because there is shared homology among some coronaviruses for the aligned KP1, CD1 and CL1 sequences, sometimes more so than SARS-CoV-2 (**Table S4**). The cross-reactive potential of the bacterial epitopes KP2, SE1 and SA1 to other coronavirus spike proteins may be of less relevance because all other coronaviruses analysed share less homology to the bacterial sequence than the SARS-CoV-2 sequence. Cross-reactive responses between the bacterial sequences and other coronaviruses may be possible but was not explored in this study.

The emergence of SARS-CoV-2 variants has the potential to impact heterologous immunity to the identified epitopes if the mutations arise at those epitopes. Mutations could arise which either increase or decrease homology and thus impact the extent of a cross-reactive T cell response. To date, no defining SNP within the major variants of concern or variants of interest has impacted the homology regions except for lineage B.1.617.2 (Delta variant) which, in a subset of sequences, contain a S:W258L mutation impacting the homology of the KP2 and SE1 peptide pairs. Since the S:W258L is not a defining SNP of the delta variant, its significance is notable but likely not widespread.

For a T cell to respond to an antigen homologue, the immunogenic homologue must be able to bind with sufficient affinity to cognate MHC class I or II. HLA binding in the context of COVID-19 has been related to disease severity as patients with mild COVID-19 presented MHCI molecules with a higher theoretical affinity than COVID-19 infected individuals with moderate or severe disease (18). Here, we show by *in silico* binding prediction analysis that pathogenic bacteria-derived peptides that share homologous sequences with SARS-CoV-2-derived peptides exhibit broad and high MHC class I and II binding affinity, which permits antigen presentation across a globally representative pool of HLA alleles (**Figures S1A, B**). These high-homology and high-binding peptides were then selected for *in vitro* co-culture analysis (**Figure 1**).

### T Cells Primed With Bacterial Peptides Have Enhanced Immune Responses to SARS-CoV-2-Peptide Homologues

Cross-reactive T cell responses were measured by TNF, IFN-γ, IL-2 and perforin expression by ICS, early T cell activation marker CD69 and a two-stain proliferation assay to delineate between a primary and secondary proliferative response. The enhanced cross-reactive responses in T cells primed with bacterial peptide, then SARS-CoV-2 restimulated compared with control primed and SARS-CoV-2 restimulated (**Figure 2**) confirms the high-homology and high and broad peptide-MHC binding affinity predictions from the *in silico* analysis (**Figures 1**, **S1A, B**). Furthermore, we show the cross-reactive peptide homologues are immunogenic as they are able to elicit broad Th1-like responses in CD4+ T cells and robust CD8+ cytotoxic responses when SARS-CoV-2 peptide or protein restimulated.

IL-2, TNF and IFN-γ cytokine responses were significantly increased in at least 5 of the 6 peptide homologue pairs in both CD4+ and CD8+ T cells (**Figures 2**, **3**). The control primed samples were not all equal to zero, which may be explained by bystander effects. IL-2, TNF and IFN-γ cytokine responses were replicated when using recombinant SARS-CoV-2 spike glycoprotein (**Figure S4**) instead of spike-derived peptide signifying the antigen-specificity of the response stems from the epitope within the 15mer of the spike glycoprotein of SARS-CoV-2, a protein which has been shown to elicit dominant T cell immune responses in COVID-19 infected individuals (22). Furthermore, these data suggest the ability of DCs to process the spike protein and present the cross-reactive epitopes of KP2, SE1 and SA1 to elicit cross-reactive T cell responses in both CD4+ and CD8+ T cells. The use of whole recombinant NSP3 protein as the antigen led to significant T cell cytotoxicity meaning cross-reactive responses using NSP3 protein could not be assessed yet T cells remained viable with NSP3 peptide. NSP3, is a papain-like protease, which is necessary for the generation of the SARS-CoV-2 replicase complex that promotes viral spread and it may be the proteolytic activity of this protein impacting T cell viability *in vitro* (54, 55). NSP3 is also known to elicit immune modulating effects by blocking transcription factors NF-κB and interferon responsive factor 3 (IRF3), which has previously been reported to affect interferons and the innate immune response to SARS-CoV and SARS-CoV-2 infection (52, 56). The immunomodulatory effects of NSP3 may also impact T cell viability in this assay.

Overall, cytokine expression patterns between individuals and peptide pairs varied, which is reflected in the complex pattern of T cell cytokine expression and phenotypes that pathogenic bacteria and SARS-CoV-2 are known to produce (10, 57). This observation is similar to previously reported COVID-19 T cell responses (57) (2).

Cytokine expression was abrogated when HLA-blocking was performed indicating the mechanism behind the T cell responses arises from TCR-dependent heterologous immunity (**Figure S5**).

In cytotoxic T cells, the target cell lysis protein, perforin, significantly increased in the bacterial peptide-primed group compared with control peptide primed across 3 homologue pairs (**Figure 3E**). The increase in perforin expression suggests cross-reactive CD8+ T cells have a heightened capacity to effect an antiviral response by target cell lysis.

Both CD8+ and CD4+ T cell activation and subsequent proliferation were measured by early activation marker CD69 surface expression and proliferation dye dilution by flow cytometry. In at least 5 of the 6 peptide homologue pairs assessed, bacterial peptide sensitised T cells developed significantly enhanced activation and proliferation upon SARS-CoV-2 restimulation (**Figures 3**, **4**). An increase in T cell activation permits clonal expansion of the cross-reactive T cells and enhancement of their effector functions which is necessary to mount an effective SARS-CoV-2 immune response. Indeed, the heightened T cell activation responses likely resulted in heightened proliferative responses shown by proliferation assay where it was seen that 3 of the 6 peptide pairs showed a significant increase in CTY^lo^CTV^lo^ proliferation when bacterial peptide-primed and SARS-CoV-2 restimulated compared with control (**Figures 4A, B**). Proliferation responses compared between control primed and bacterial peptide-primed showed T cells were able to proliferate even in the control samples, which could be explained by TCR-independent effects such as bystander activation (**Figure 4C**). However, compared to control primed, the samples which underwent bacterial peptide priming proliferated more (CTY^lo^CTV^lo^).The proliferation assay with two proliferation dyes was set up to differentiate T cells proliferating in response to 1) bacterial peptide priming and not SARS-CoV-2 restimulation (CTY^lo^CTV^hi^), 2) both bacterial peptide priming and SARS-CoV-2 restimulation (CTY^lo^CTV^lo^), 3) not bacterial peptide priming but SARS-CoV-2 restimulation (CTY^hi^CTV^lo^) or 4) neither to bacterial peptide priming nor SARS-CoV-2 restimulation (CTY^hi^CTV^hi^) (**Figure 4C**). The proliferation assay showed most T cells in response to bacterial peptide priming had proliferated suggesting a proportion of them were clonally expanded, bacterial-peptide specific T cells with the rest proliferating by TCR-independent effects such as bystander activation (**Figure S6**).

*In vitro* assays were established with CD3+ T cells and dendritic cells as opposed to PBMCs to minimise the immunomodulatory effects other immune cells may have on the DC-T cell interaction and generate a greater resolution of the TCR-dependent responses. Due to the nature of the assay setup, not all T cells are expected to be specific for the bacterial peptide after priming as some T cells would be present after priming whose TCR cannot recognise the bacterial peptide. Upon restimulation, bystander activation has the potential to activate such non-specific T cells and contribute to the cross-reactive response. Furthermore, given there is a degree of sequence homology between the bacterial epitopes, SARS-CoV-2 epitopes and other coronavirus epitopes (Tab. S4), it is possible that some of the responses are heightened by pre-existing memory T cells to other coronaviruses or the bacterial infections. Given the samples were negative for SARS-CoV-2 spike antibodies and donors reported no previous infection with SARS-CoV-2, pre-existing SARS-CoV-2 memory T cells in the *in vitro* assays are not present.

Overall, pre-exposure to the 6 identified epitopes from pathogenic bacteria produce T cells which are able to cross-react with their corresponding SARS-CoV-2 homologous epitope. In the context of human disease, these bacterial pathogens may seed T cell memory which has the capacity to generate heightened T cell responses upon SARS-CoV-2 infection. To date, there have not been studies pairing historical infection with the aforementioned bacterial pathogens and COVID-19 severity. Such studies would be of benefit in elucidating the extent to which such cross-reactive T cells play in COVID-19 severity outcomes.

### T Cells Exposed to SARS-CoV-2 Vaccination Have Enhanced Immune Responses to Bacterial-Peptide Homologues

Cytokine responses tracked between individuals before and after SARS-CoV-2 vaccination showed a significant upregulation of proinflammatory cytokines IFN-γ; TNF and IL-2 across both CD4+ and CD8+ T cells. This indicates SARS-CoV-2 vaccination, encoding spike protein, is able to seed T cell memory which has the capacity to cross-react with SA1, SE1 and KP2 epitopes of bacterial origin. Although responses were generally low in pre-vaccinated samples, there was a variation between individuals in the degree of their pre-vaccinated T cell response to the bacterial epitopes, which may be due to T cell memory seeded from prior exposure to the bacterial pathogens. SARS-CoV-2 peptide cytokine responses tracked between individuals were demonstrably higher post-vaccination (**Figure S8**), which indicates successful vaccination seeding the SARS-CoV-2 spike protein T cell memory against the particular epitopes, namely S_245-268_, S_255-269_ and S_1110-1114_ and supports their previously reported immunodominance (22). Cytokine responses compared between post-vaccinated samples stimulated with bacterial or SARS-CoV-2 peptides showed the SARS-CoV-2 peptide stimulated samples were slightly higher than the bacterial peptide stimulated sample. This can be explained by the incomplete homology between bacterial and SARS-CoV-2 epitopes yet highlights that most of the T cells specific for the spike protein were able to cross-react with the bacterial peptide homologue.

The T cells responding to the bacterial epitopes were approximately 80% T memory phenotype (**Figure S7A**). Such a skewing of a memory response as opposed to a primary T cell response from the naïve T cell subset, and the significant increase in cytokine responses post-vaccination suggest the antigen-specific cells arose from prior exposure to the antigen homologue, most likely from SARS-CoV-2 vaccination.

T cell proliferation responses also correlated with the cytokine responses such that there was a significant increase in CD4+ and CD8+ T cell proliferation present in the post-vaccinated samples compared with pre-vaccinated when presented with the bacterial homologue peptides SA1, SE1 and KP2. This is due to the memory T cells generated by SARS-CoV-2 vaccination cross reacting with the bacterial epitopes, due to shared homology, and in turn activating the T cells to undergo clonal expansion. Such cross-reactive proliferative responses may have a role in combatting such bacterial infections in individuals who received SARS-CoV-2 vaccine. However, the presence and extent of any such protection remains to be determined.

Activation-induced marker (AIM) assays allow for the identification of TCR-dependent, antigen-specific T cell responses by analysis of the co-expression of T cell activation markers. It has been shown that bystander activation has little impact on the co-expression of such markers (21). AIM+ T cells have been used to characterise immunodominant T cell epitopes to SARS-CoV-2 and these immunodominant spike epitopes overlap with the homologous sequences in SA1, SE1 and KP2 (22). Our results indicate SA1-, SE1- and KP2-specific T memory cells were increased post-SARS-CoV-2 vaccination. The proposed mechanism for this increase is the homology shared between these bacterial epitopes and the immunodominant T cell epitopes from the spike protein. A subset of the memory T cells created in response to SARS-CoV-2 vaccination have the capacity, through their TCR, to recognise the pathogenic bacterial homologue sequences found in SA1, SE1 and KP2.

Three separate methods of tracking cross-reactive T cell responses pre- and post-SARS-CoV-2 vaccination were explored, namely ICS, proliferation assay and AIM assay and all 3 assays indicated that SARS-CoV-2 vaccination enhances the cross-reactive T cell repertoire to the bacterial epitopes found in SA1, SE1 and KP2. By having such T cell memory against not only SARS-CoV-2 but additionally, through cross reactive TCRs*, K. pneumoniae, S. enteritidis, E. faecalis, S. aureus* and *K. grimontii*, the SARS-CoV-2 vaccine has the potential to confer added T cell mediated immunity to these bacterial pathogens yet this requires further clinical correlations. The mechanism of such peptide homologues acting as cross-reactive B cell epitopes may also exist and warrants further investigation.

### Limitations of the Study

It is important to consider these homologous epitopes comprise a small subset of the total number of epitopes possible from these pathogens and accordingly the extent of any cross-reactive effects against such pathogens remains to be determined. Delineation of the critical amino acids contributing to the observed cross-reactivity would assist in further characterising the epitopes. Assays to remove bystander effects would also assist in better resolving the cross-reactive immune responses. Further studies are needed to identify and characterise whether cross-reactivity results in any cross-protection or increased disease risk, particularly assessing disease severity outcomes in patients with such bacterial infections or COVID-19.

## Conclusion

We have identified 6 epitopes originating from pathogenic bacteria that share homology with SARS-CoV-2 spike protein (3 epitopes) or NSP3 protein (3 epitopes). Collectively, the high *in silico* binding affinity of these bacterial epitopes across a globally representative set of HLA alleles, their ability to cross-react *in vitro* with SARS-CoV-2 homologous epitopes and finally the *ex vivo* increase in frequency and responsiveness of cross-reactive T cells to the spike protein homologues after SARS-CoV-2 vaccination, all indicate the 6 epitopes constitute immunodominant epitopes capable of cross-reacting between pathogenic bacteria and SARS-CoV-2 infection or vaccination. The significance of these findings could work both ways. Prior exposure to these bacterial pathogens may seed T cell memory and provide a degree of heterologous immunity during SARS-CoV-2 infection or vaccination. Conversely, SARS-CoV-2 infection or vaccination may seed T cell memory that produces a degree of heterologous immunity against bacterial pathogens. Further insight into such a mechanism may assist in unravelling the unpredictable heterogeneity of SARS-CoV-2 clinical manifestations.

## Data Availability Statement

The original contributions presented in the study are included in the article/**Supplementary Material**. Further inquiries can be directed to the corresponding author.

## Ethics Statement

The studies involving human participants were reviewed and approved by Monash University Human Research Ethics Committee (project ID 25834). The patients/participants provided their written informed consent to participate in this study.

## Author Contributions 

PE designed the project, performed the experiments, analysed data and wrote the manuscript. BN, AY, and JC performed the experiments, analysed data and provided intellectual input. WW analysed data. RC analysed the data, wrote the manuscript and provided intellectual input, JM, YT, PG, and SH analysed data and provided intellectual input. JO designed the project, analysed data and wrote the manuscript. All authors contributed to the article and approved the submitted version.

## Funding

The project was funded by Monash Health Foundation COVID-19 Research Fund Grant.

## Conflict of Interest

The authors declare that the research was conducted in the absence of any commercial or financial relationships that could be construed as a potential conflict of interest.

## Publisher’s Note

All claims expressed in this article are solely those of the authors and do not necessarily represent those of their affiliated organizations, or those of the publisher, the editors and the reviewers. Any product that may be evaluated in this article, or claim that may be made by its manufacturer, is not guaranteed or endorsed by the publisher.
