# Supplementary material for: Heterologous Immunity Between SARS-CoV-2 and Pathogenic Bacteria
Source: Front Immunol. 2022 Jan 27;13:821595. doi: 10.3389/fimmu.2022.821595 (PMC8829141; doi:10.3389/fimmu.2022.821595)
Supplement: Supplementary file 1 [file DataSheet_1.pdf]

Supplementary Material

Heterologous immunity between SARS-CoV-2 and pathogenic bacteria

Peter J. Eggenhuizen[1], Boaz H. Ng[1], Janet Chang[1], Rachel M.Y. Cheong[1], Anusha Yellapragada[1], Wey Y. Wong[1], Yi Tian Ting[1], Julie A. Monk[1], Poh-Yi Gan[1,2], Stephen R. Holdsworth[1,2], Joshua D. Ooi*[1]

[1]Centre for Inflammatory Diseases, Department of Medicine, Monash Medical Centre, School of Clinical Sciences, Monash University, Clayton, Victoria 3168 Australia.

[2]Department of Immunology, Monash Health, Monash Medical Centre, Clayton, Victoria 3168 Australia.

*** Correspondence:**
Corresponding Author
joshua.ooi@monash.edu

1.1 Supplementary Tables

Table S1 | Sequence homology between pathogenic bacteria and SARS-CoV-2 9mers

Peptide pair	Bacterial peptide	SARS-CoV-2 peptide	% Similarity	Peptide pair	Bacterial peptide	SARS-CoV-2 peptide	% Similarity
KP1	DVIVNAANP	TVVVNAANV	77.8	KP2	GSVVLPAGA	SSSGWTAGA	44.4
	VIVNAANPS	VVVNAANVY	77.8		SVVLPAGAA	SSGWTAGAA	55.6
	IVNAANPSL	VVNAANVYL	77.8		VVLPAGAAA	SGWTAGAAA	55.6
	VNAANPSLL	VNAANVYLK	66.7		VLPAGAAAY	GWTAGAAAY	66.7
	NAANPSLLG	NAANVYLKH	55.6		LPAGAAAYY	WTAGAAAYY	77.8
	AANPSLLGG	AANVYLKHG	55.6		PAGAAAYYR	TAGAAAYYV	77.8
	ANPSLLGGG	ANVYLKHGG	55.6		AGAAAYYRR	AGAAAYYVG	77.8
SE1	SEGWTVKQA	SSGWTAGAA	55.6	SA1	KSMFVTRKN	THWFVTQRN	66.7
	EGWTVKQAA	SGWTAGAAA	55.6		SMFVTRKNY	HWFVTQRNF	77.8
	GWTVKQAAY	GWTAGAAAY	66.7		MFVTRKNYY	WFVTQRNFY	88.9
	WTVKQAAYY	WTAGAAAYY	66.7		FVTRKNYYE	FVTQRNFYE	100
	TVKQAAYYV	TAGAAAYYV	66.7		VTRKNYYED	VTQRNFYEP	88.9
	VKQAAYYVG	AGAAAYYVG	66.7		TRKNYYEDE	TQRNFYEPQ	88.9
	KQAAYYVGY	GAAAYYVGY	77.8		RKNYYEDEV	QRNFYEPQI	88.9
CD1	DAIVNAANS	TVVVNAANV	66.7	CL1	DAIVNAANG	TVVVNAANV	66.7
	AIVNAANST	VVVNAANVY	66.7		AIVNAANGM	VVVNAANVY	66.7
	IVNAANSTL	VVNAANVYL	77.8		IVNAANGML	VVNAANVYL	77.8
	VNAANSTLL	VNAANVYLK	66.7		VNAANGMLK	VNAANVYLK	77.8
	NAANSTLLG	NAANVYLKH	55.6		NAANGMLKH	NAANVYLKH	77.8
	AANSTLLGG	AANVYLKHG	55.6		AANGMLKHG	AANVYLKHG	77.8
	ANSTLLGGG	ANVYLKHGG	55.6		ANGMLKHGG	ANVYLKHGG	77.8

Table S2| HLA-typed donor alleles used in this study.

Donor ID	HLA-A	HLA-B	HLA-C	HLA-DRB1	HLA-DRB3	HLA-DRB4	HLA-DPB1	HLA-DPA1	HLA-DQB1	HLA-DQA1
H2	01:01, 32:01	14:01, 44;02	05:01, 08:02	07:01, 11:01	02:02	01:01	04:02	01:03	02:02, 03:01	02:01, 05:05
H3	02:07, 32:01	48:01, 52:01	08:03, 12:02	09:01, 14:05	02:02:01G	01:03	02:01, 02:02	01:03, 02:02	03:03, 05:03	01:04, 03;02
H4	02:01, 11:01	14:02, 35:01	04:01, 08:02	01:02, 14:54	02:02		04:01, 16:01	01:03	05:01, 05:03	01:01, 01:04
H6				03,04						
H7	03:01, 11:01	38:01, 54:01	07:02, 12:03	04:05, 07:01		01:03	02:02:01G, 05:01:01G	02:02,	02:02/156/163N, 04:01	02:01, 03:03
H8	11:01	13:01, 40:01	03:04, 07:02	08:09, 12:02	03:01		03:01, 05:01	01:03, 02:02	03:01, 04:02	04:01, 06:01
H9				09,16						
H11				12						
H13	01:01:01, 02:01:01	07:02:01, 18:01:01	07:01:01, 07:02:01	04:01:01, 04:04:01		01:03:01	03:01:01, 06:01:01	01:03:01	03:02:01G	03:01:01
H14	30:01, 31:01	13:02, 40:01	03:04, 06:02	07:01, 12:01/10	01:01	01:03	05:01	02:02	02:02/156/163N, 03:01/276N	02:01, 05:08

Table S3| Summary of pathogenic bacteria sharing homology with SARS-CoV-2

Bacterium	Pathology	Isolated from COVID-19 lower airways in Sulaiman, et al? (1)	T cell involvement
K. pneumoniae	Pneumonia, urinary tract infections, bloodstream infections (2)	Yes	Yes (3)
K. grimontii	Bacteraemia, wound infections, and antibiotic-associated haemorrhagic colitis (4)	No	NA
E. coli	Gastroenteritis, acute urinary tract infections, neonatal meningitis and sepsis, and acute enteritis (5)	Yes	Yes (6)
S. Enteritidis	Inflammatory diarrhoea and gastroenteritis (7)	No	Yes (8)
E. faecalis	Urinary tract infections, endocarditis, and bacteraemia (9)	No	Yes (10)
S. aureus	Bacteraemia, infective endocarditis, and skin infections (11)	Yes	Yes (12)
C. freundii	Urinary tract infections and bacteraemia (13)	No	NA
C. difficile	Mild diarrhoea and pseudomembranous colitis (14)	Yes	Yes (15)
C. haemolyticum	Liver, bone marrow, and bloodstream infections (rare) (16)	No	NA
C. novyi	Gas gangrene and soft tissue infections (17)	No	NA

Table S4| Sequence alignment of bacterial peptides and human coronaviruses. Amino acid identity (red), similarity (yellow) and dissimilarity (grey) between the bacterial 15mer sequences (black) from Fig. 1 and human coronaviruses. SARS-CoV-2 – original reference sequence of Severe acute respiratory syndrome coronavirus 2. B.1.617.2 – SARS-CoV-2 Delta variant of concern. BA.1 – SARS-CoV-2 Omicron variant of concern. SARS-CoV - Severe acute respiratory syndrome coronavirus 1. MERS-CoV - Middle East respiratory syndrome coronavirus. OC43 and HKU1 – human betacoronaviruses. NL63 and 229E – human alphacoronaviruses.

KP1	D	V	I	V	N	A	A	N	P	S	L	L	G	G	G
SARS-CoV-2	T	V	V	V	N	A	A	N	V	Y	L	K	H	G	G
B.1.617.2	T	V	V	V	N	A	A	N	V	Y	L	K	H	G	G
BA.1	T	V	V	V	N	A	A	N	V	Y	L	K	H	G	G
SARS-CoV	S	V	I	V	N	A	A	N	I	H	L	K	H	G	G
MERS-CoV	E	V	L	V	N	A	A	N	T	H	L	K	H	G	G
OC43	D	V	V	V	N	P	A	N	G	H	M	A	H	G	G
HKU1	D	V	I	V	N	P	A	N	G	H	M	L	H	G	G
NL63	D	F	V	V	N	A	A	N	E	N	L	L	H	G	G
229E	D	F	I	V	N	A	A	N	E	N	L	A	H	G	G

KP2	G	S	V	V	L	P	A	G	A	A	A	Y	Y	R	R
SARS-CoV-2	S	S	S	G	W	T	A	G	A	A	A	Y	Y	V	G
B.1.617.2	S	S	S	G	W	T	A	G	A	A	A	Y	Y	V	G
BA.1	S	S	S	G	W	T	A	G	A	A	A	Y	Y	V	G
SARS-CoV	Q	D	I	W	G	T	S	A	A	A	A	Y	F	V	G
MERS-CoV	G	S	V	V	E	Q	A	E	G	V	E	C	D	F	S
OC43	G	S	V	L	L	S	R	L	W	F	K	P	P	F	L
HKU1	G	Q	G	I	F	K	E	V	S	A	V	Y	Y	N	S
NL63	G	S	C	N	F	P	L	E	A	T	W	H	Y	T	S
229E	G	I	M	V	L	P	G	V	A	D	A	E	R	M	A

SE1	S	E	G	W	T	V	K	Q	A	A	Y	Y	V	G	Y
SARS-CoV-2	S	S	G	W	T	A	G	A	A	A	Y	Y	V	G	Y
B.1.617.2	S	S	G	W	T	A	G	A	A	A	Y	Y	V	G	Y
BA.1	S	S	G	W	T	A	G	A	A	A	Y	Y	V	G	Y
SARS-CoV	Q	D	I	W	G	T	S	A	A	A	Y	F	V	G	Y
MERS-CoV	I	Q	S	D	R	K	A	W	A	A	F	Y	V	Y	K
OC43	N	N	T	W	M	Y	T	G	S	G	Y	Y	Y	P	E
HKU1	N	D	H	W	M	F	T	G	S	S	Y	Y	Y	P	E
NL63	L	S	I	A	D	L	A	C	A	Q	Y	Y	N	G	I
229E	T	S	H	F	T	T	K	Y	V	A	V	Y	A	N	V

SA1	K	S	M	F	V	T	R	K	N	Y	Y	E	D	E	V
SARS-CoV-2	T	H	W	F	V	T	Q	R	N	F	Y	E	P	Q	I
B.1.617.2	T	H	W	F	V	T	Q	R	N	F	Y	E	P	Q	I
BA.1	T	H	W	F	V	T	Q	R	N	F	Y	E	P	Q	I
SARS-CoV	N	S	W	F	I	T	Q	R	N	F	F	S	P	Q	I
MERS-CoV	P	C	T	F	M	Y	T	Y	N	I	T	E	D	E	I
OC43	S	S	T	W	N	K	R	F	G	F	I	E	D	S	V
HKU1	R	S	G	F	L	Q	S	S	N	Y	K	I	D	T	T
NL63	G	S	A	F	A	L	H	T	G	Y	Y	D	A	N	Q
229E	G	C	C	E	S	T	K	L	P	Y	Y	D	V	E	K

CD1	D	A	I	V	N	A	A	N	S	T	L	L	G	G	G
SARS-CoV-2	T	V	V	V	N	A	A	N	V	Y	L	K	H	G	G
B.1.617.2	T	V	V	V	N	A	A	N	V	Y	L	K	H	G	G
BA.1	T	V	V	V	N	A	A	N	V	Y	L	K	H	G	G
SARS-CoV	M	V	I	V	N	A	A	N	I	H	L	K	H	G	G
MERS-CoV	S	V	L	V	N	A	A	N	T	H	L	K	H	G	G
OC43	E	V	V	V	N	P	A	N	G	H	M	A	H	G	G
HKU1	D	V	I	V	N	P	A	N	G	H	M	L	H	G	G
NL63	D	F	V	V	N	A	A	N	E	N	L	L	H	G	G
229E	D	F	I	V	N	A	A	N	E	N	L	A	H	G	G

CL1	D	A	I	V	N	A	A	N	G	M	L	K	H	G	G
SARS-CoV-2	T	V	V	V	N	A	A	N	V	Y	L	K	H	G	G
B.1.617.2	T	V	V	V	N	A	A	N	V	Y	L	K	H	G	G
BA.1	T	V	V	V	N	A	A	N	V	Y	L	K	H	G	G
SARS-CoV	M	V	I	V	N	A	A	N	I	H	L	K	H	G	G
MERS-CoV	S	V	L	V	N	A	A	N	T	H	L	K	H	G	G
OC43	E	V	V	V	N	P	A	N	G	H	M	A	H	G	G
HKU1	D	V	I	V	N	P	A	N	G	H	M	L	H	G	G
NL63	D	F	V	V	N	A	A	N	E	N	L	L	H	G	G
229E	D	F	I	V	N	A	A	N	E	N	L	A	H	G	G

1.2 Supplementary Figures

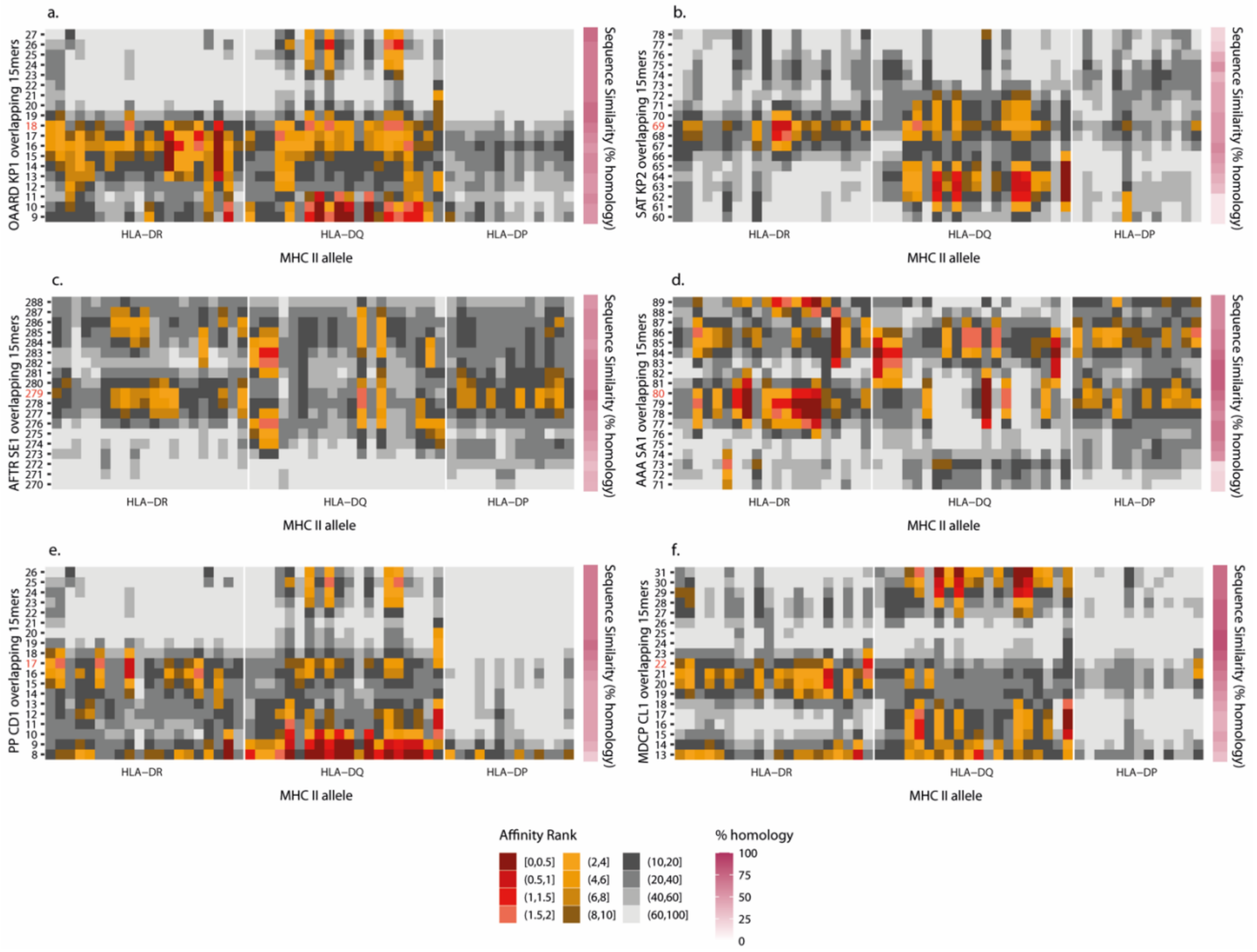

Figure S1a| Regions of bacterial-SARS-CoV-2 homology exhibit broad HLAII binding

Affinity rank score of bacterial 15mers overlapping by 1 amino acid across the region of shared homology between bacteria and SARS-CoV-2. Hotspots of high affinity overlap broadly with high regions of homology. Red gradient; strong peptide-MHC binder (affinity rank ≤ 2). Yellow gradient; peptide-MHC binder (affinity rank > 2 and ≤ 10). Grey gradient; non-binder (affinity rank < 10). Y-axis; number indicates the amino acid sequence start number of the respective 15mer. Red number indicates the 15mers analysed in this study. X axis; MHC class II alleles grouped into HLA-DR (n=20), HLA-DQ (n=22) and HLA-DP (n=15) isotype. The selected alleles are globally representative and include all alleles from HLA-typed donors used in this study. Pink gradient; Pairwise percent sequence similarity between bacteria and SARS-CoV-2 15mers. (A) OAARD - O-acetyl-ADP-ribose deacetylase affinity rank binding scores, (B) SAT - serine acetyltransferase affinity rank binding scores, (C) AFTR - AraC family transcriptional regulator affinity rank binding scores, (D) AFA - AAA family ATPase affinity rank binding scores, (E) PP - Putative phosphatase affinity rank binding scores, (F) MDCP - Macro domain containing protein affinity rank binding scores.

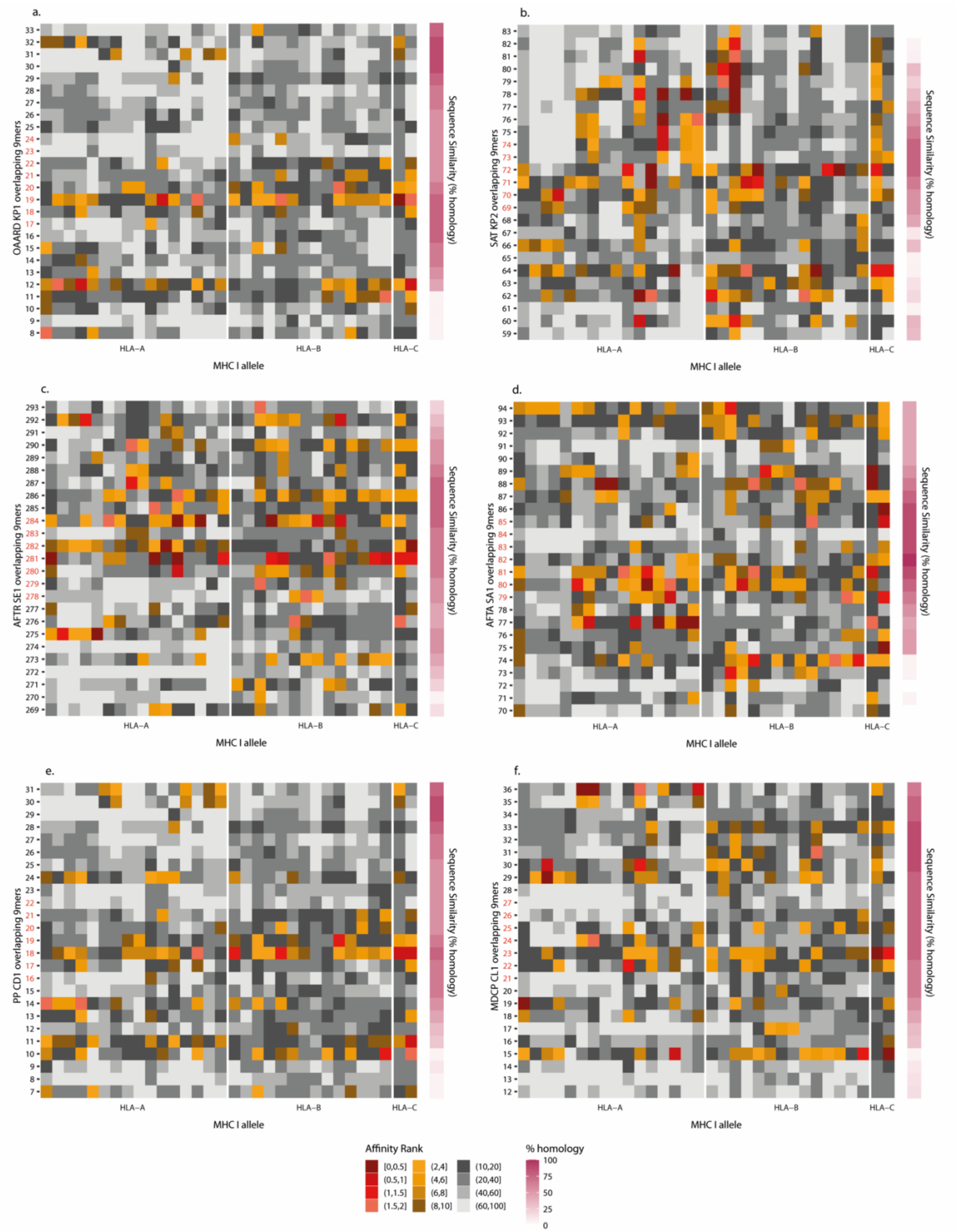
a.
OAARD KP1 overlapping 9mers
b.
SAT KP2 overlapping 9mers
c.
AFTR SE1 overlapping 9mers
d.
AFTA SA1 overlapping 9mers
e.
PP CD1 overlapping 9mers
f.
MDCP CL1 overlapping 9mers
HLA−A
HLA−B
HLA−C
MHC I allele
Sequence Similarity (% homology)
Affinity Rank
[0,0.5]
(0.5,1]
(1,1.5]
(1.5,2]
(2,4]
(4,6]
(6,8]
(8,10]
(10,20]
(20,40]
(40,60]
(60,100]
% homology
100
75
50
25
0

Figure S1b| Regions of bacterial-SARS-CoV-2 homology exhibit broad HLA-I binding Affinity rank score of bacterial 9mers overlapping by 1 amino acid across the region of shared homology between bacteria and SARS-CoV-2. Red gradient; strong peptide-MHC binder (affinity rank ≤ 2). Yellow gradient; peptide-MHC binder (affinity rank > 2 and ≤ 10). Grey gradient; non-binder (affinity rank < 10). Y-axis; number indicates the amino acid sequence start number of the respective 9mer. Red number indicates the overlapping 9mers contained within the 15mers analysed in this study. X-axis; MHC class I alleles grouped into HLA-A (n=16), HLA-B (n=14) and HLA-C (n=2) isotype. These alleles selected are globally representative and include the alleles from HLA-typed donors used in this study. Pink gradient; Pairwise percent sequence similarity between bacteria and SARS-CoV-2 9mers. (A) OAARD - O-acetyl-ADP-ribose deacetylase affinity rank binding scores, (B) SAT - serine acetyltransferase affinity rank binding scores, (C) AFTR - AraC family transcriptional regulator affinity rank binding scores, (D) AFA - AAA family ATPase affinity rank binding scores, (E) PP - Putative phosphatase affinity rank binding scores, (F) MDCP - Macro domain containing protein affinity rank binding scores.

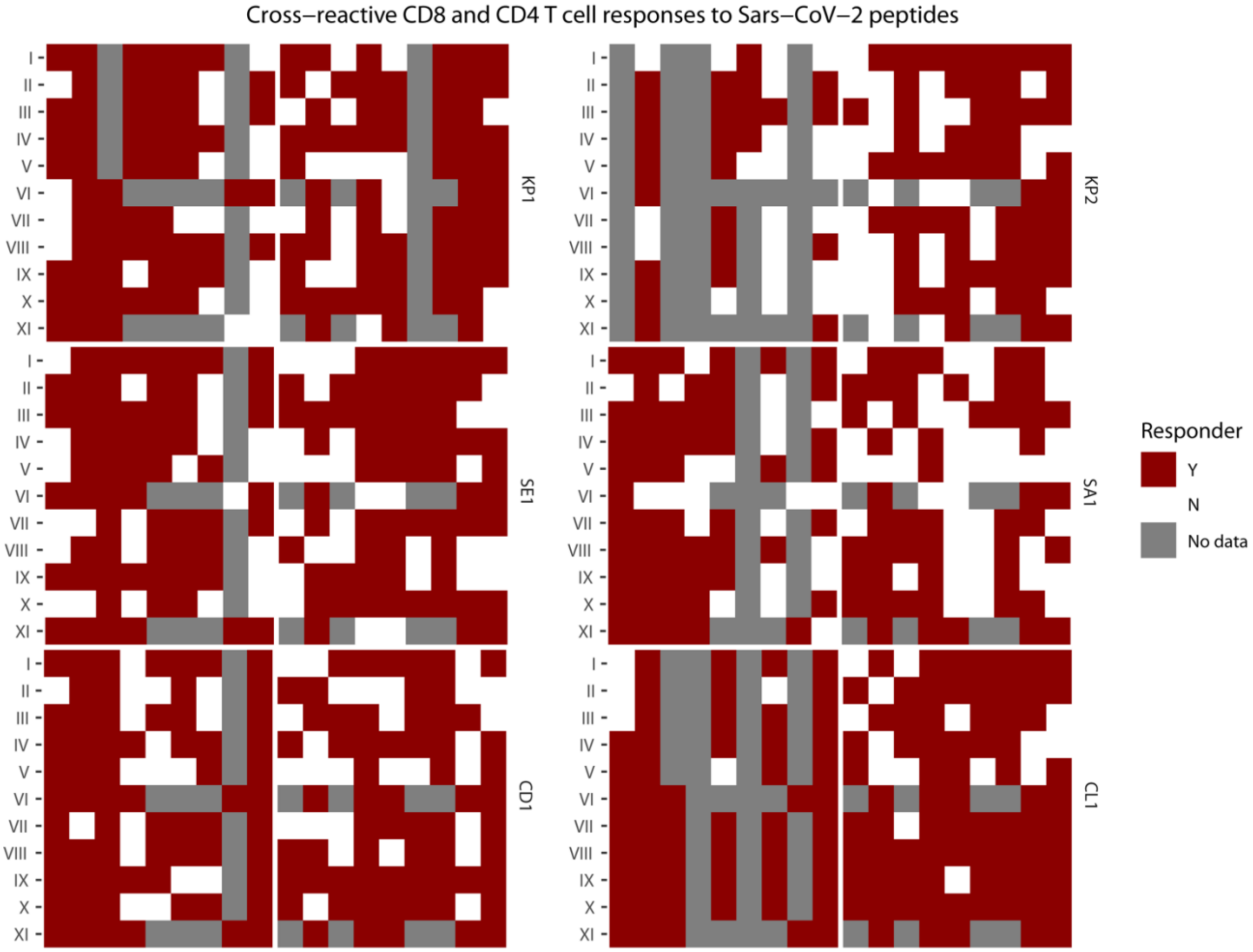

Figure S2| Pathogenic bacterial peptides induce broad cross-reactive T cell responses to SARS-CoV-2 across individuals.

Heat map of individuals (n=18) representing global HLA coverage shows improved SARS-CoV-2 T cell responses when pre-stimulated with bacterial peptide. Individual donor T cell responses to the 6 peptide pairs (KP1, KP2, SE1, SA1, CD1, CL1) across 11 parameters (i-xi) determined by flow cytometry. i- CD8+IFN-γ, ii- CD8+TNF, iii- CD8+IL-2, iv- CD8+CD69, v- CD8+Perforin, vi- CD8+proliferation, vii- CD4+IFN-γ, viii- CD4+TNF, ix- CD4+IL-2, x- CD4+CD69, xi- CD4+proliferation. A responder (red) is defined as showing a positive response after subtraction of the control-primed response to SARS-CoV-2. A non-responder (white) is defined as showing no positive staining after subtraction of the control response. Grey –no data.

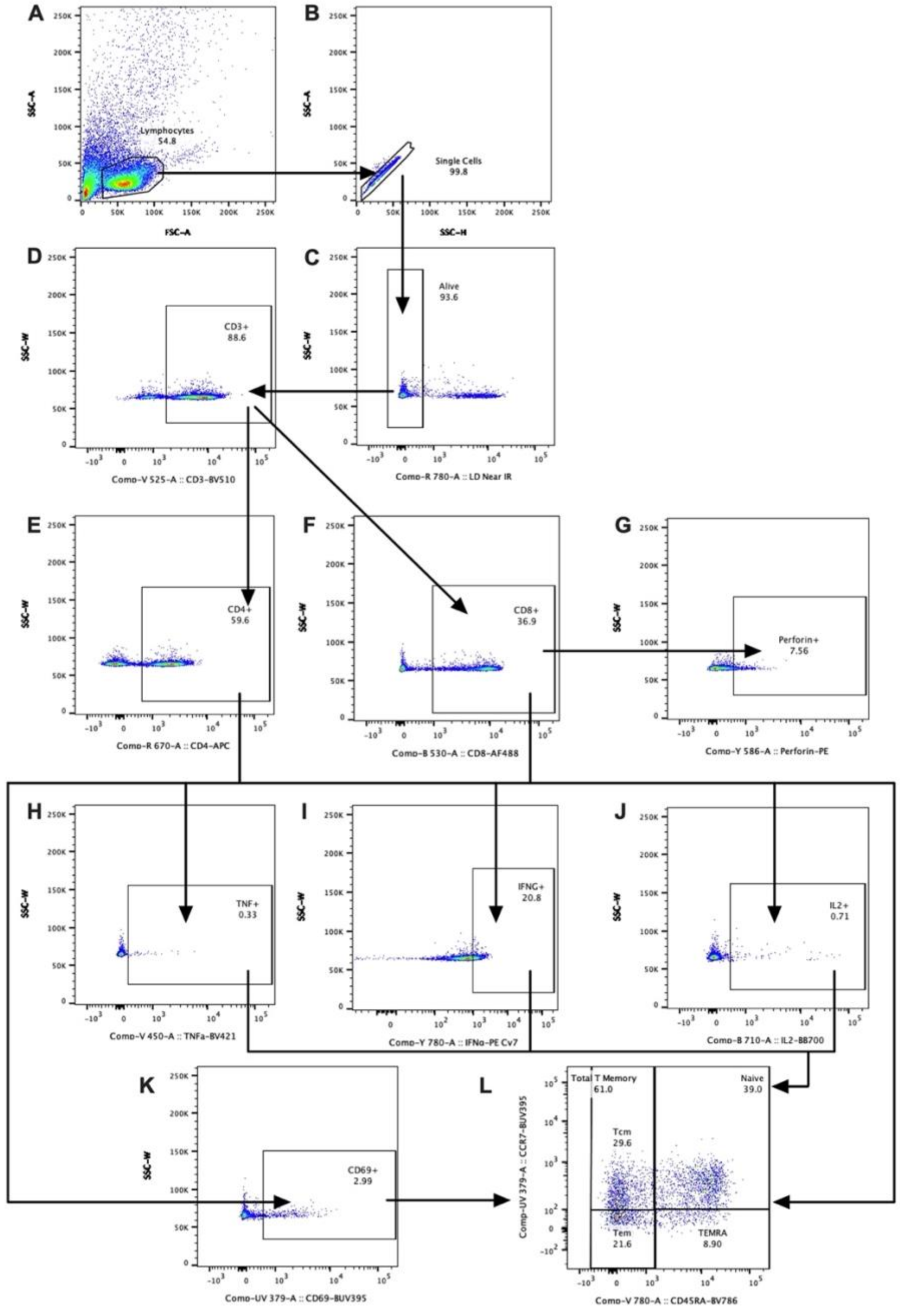

Figure S3a| Intracellular Cytokine Staining flow cytometry gating strategy

Intracellular cytokine staining (ICS) flow cytometry gating performed by (A) forward scatter area (FSC-A) vs. side scatter area (SSC-A) selecting the lymphocytes, (B) Side scatter height (SSC-H) vs. SSC-A selecting the singlets, (C) Viability dye (LD near IR) vs. side scatter width (SSC-W) selecting the alive cells, (D) CD3 vs. SSC-W selecting pan T cells, (E) CD4 vs. SSC-W selecting the CD4+ T cells. (F) CD8 vs. SSC-W selecting the CD8+ T cells. (G) Perforin vs. SSC-W selecting the Perforin positive cells of the CD8+ parent population. (H) TNF vs. SSC-W. (I) IFN-γ vs. SSC-W. (J) IL-2 vs. SSC-W. (K) CD69 vs. SSC-W. (L) In some samples, CD69-BUV395 was replaced with CCR7-BUV395 and Perforin-PE was replaced with CD69-PE. Then, CD45RA vs. CCR7 quadrant gating of Tcm, Tem, TEMRA, T naïve and T memory subsets was performed to assess T memory/naïve phenotypes. Gating was determined based on fluorescence minus one (FMO) controls.

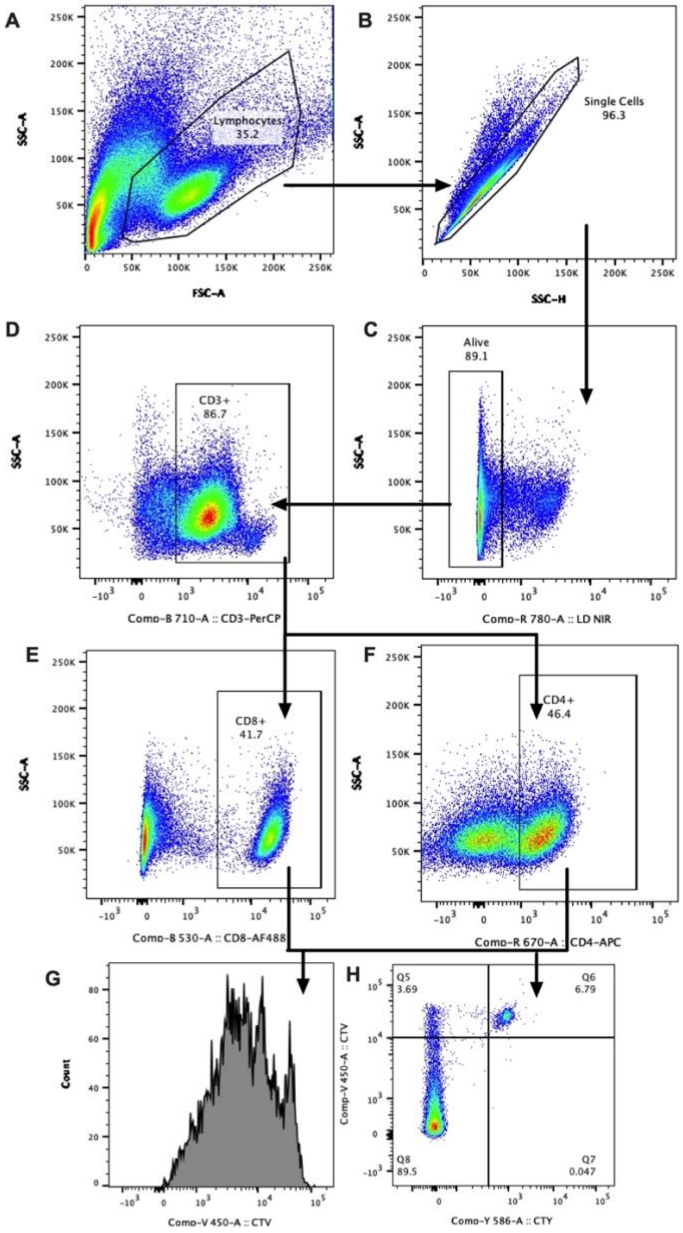

Figure S3b| T cell proliferation flow cytometry gating strategy

T cell proliferation gating strategy performed by (A) forward scatter area (FSC-A) vs. side scatter area (SSC-A) selecting the lymphocytes. (B) Side scatter height (SSC-H) vs. SSC-A selecting the singlets. (C) Viability dye (LD near IR) vs. SSC-A selecting the alive cells. d) CD3 vs. SSC-A selecting pan T cells. (E) CD8 vs. SSC-A selecting the CD8+ T cells. (F) CD4 vs. SSC-A selecting the CD4+ T cells. (G) For pre/post vaccination experiments, proliferation dye CellTrace Violet (CTV) histogram. (H) For *in vitro* cross-reactivity experiment, CellTrace Yellow (CTY) vs. CTV were quadrant gated based on proliferation after priming and restimulation. Q5 – CTY^{low} CTV^{high} T cells that proliferated after priming but not after restimulation. Q6 – CTY^{high} CTV^{high} T cells that did not proliferate after priming or restimulation. Q7 CTY^{high} CTV^{low} T cells that did not proliferate after priming but proliferated after restimulation. Q8 – CTY^{low} CTV^{low} T cells that proliferated both after priming and restimulation.

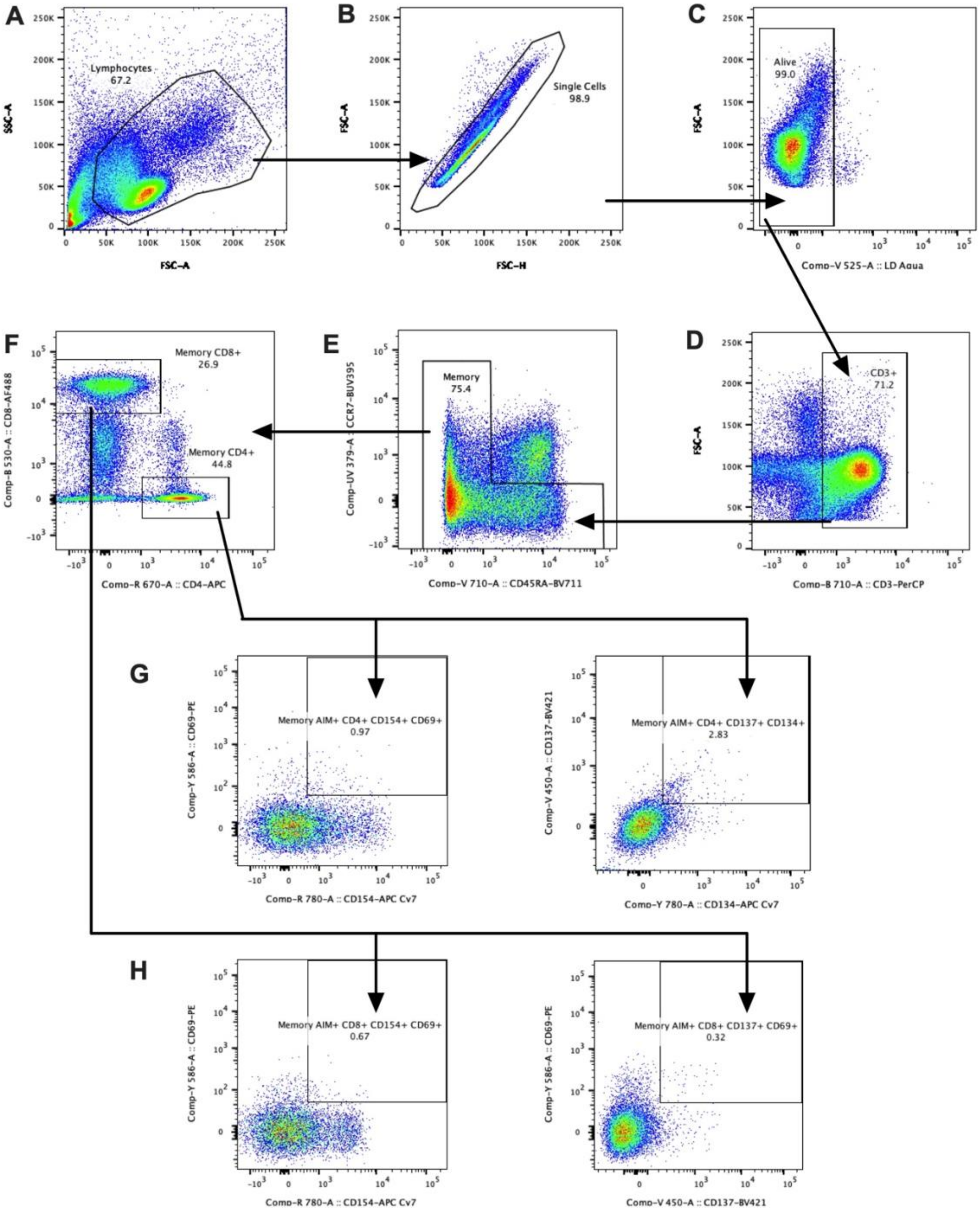

Figure S3c| Memory AIM flow cytometry gating strategy

Activation-induced marker (AIM) gating strategy performed by (A) forward scatter area (FSC-A) vs. side scatter area (SSC-A) selecting the lymphocytes. (B) Forward scatter height (FSC-H) vs. FSC-A selecting the singlets. (C) Viability dye (LD Aqua) vs. FSC-A selecting the alive cells. (D) CD3 vs. FSC-A selecting pan T cells. (E) CD45RA vs. CCR7 selecting the T memory cells. (F) CD4 vs CD8 selecting the CD4+ and CD8+ memory T cells. (G) CD154 vs. CD69 and CD134 vs. CD137 selecting the AIM+ CD4+ memory T cells. (H) CD154 vs. CD69 and CD137 vs. CD69 selecting the AIM+ CD8+ memory T cells.

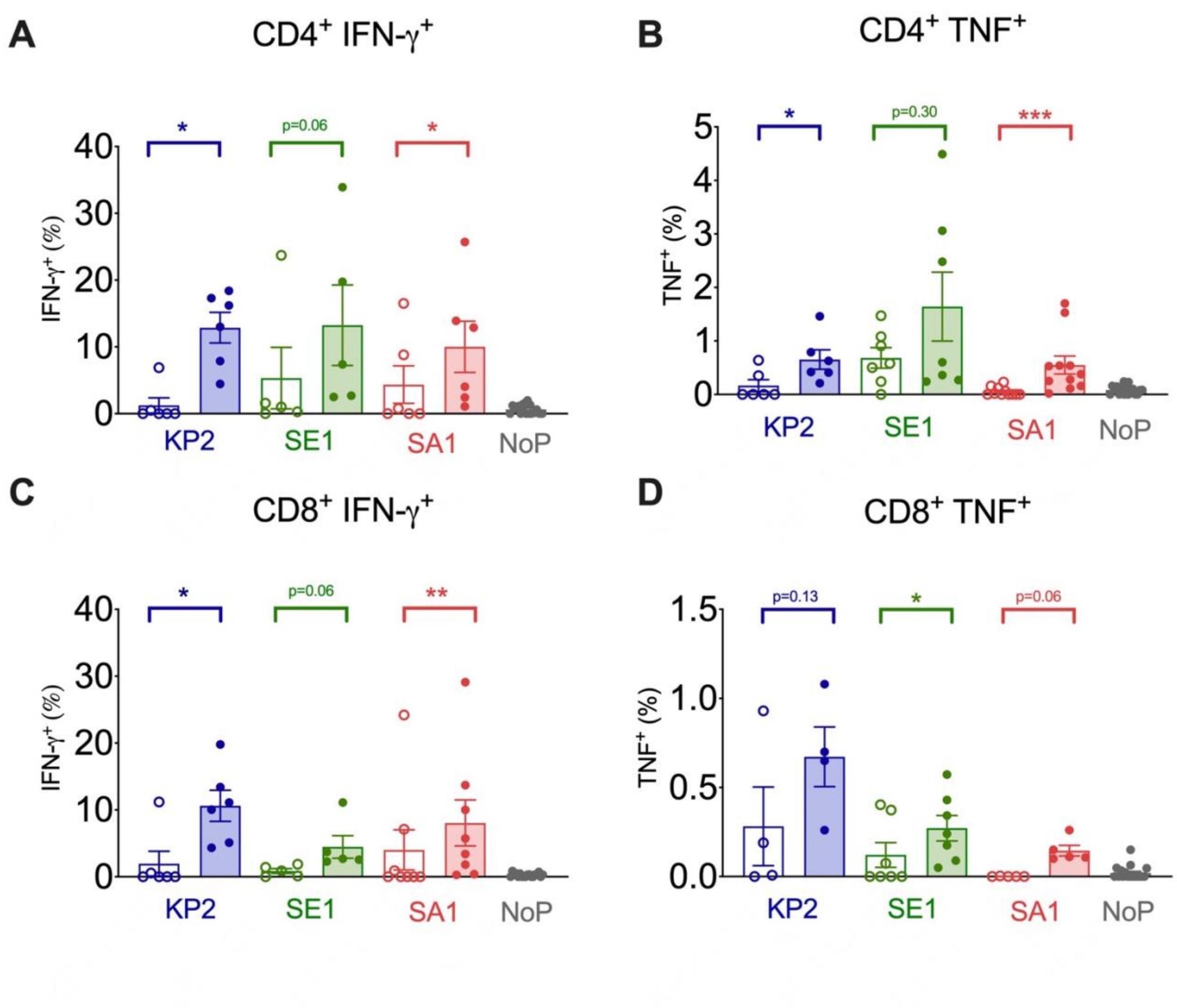

Figure S4| Bacterial peptide priming enhances T cell responses against recombinant SARS-CoV-2 spike protein

Pathogenic bacterial peptide-primed T cells restimulated with SARS-CoV-2-recombinant protein cytokine responses measured by intracellular cytokine staining. Unshaded bars- Control primed (irrelevant peptide, PVSKMRMATPLLMQA), then restimulated with SARS-CoV-2 spike protein. Shaded bars– bacterial peptide primed (KP2, SE1 or SA1) then SARS-CoV-2 spike protein restimulated. NoP – no peptide or protein negative control. $*P < 0.05$, $**P < 0.01$, $***P < 0.001$ by Wilcoxon-matched-pairs-signed-rank test. (A) $CD4^{+}IFN\text{-}\gamma^{+}$ responses (n= 5-6). (B) $CD4^{+}TNF^{+}$ responses (n= 6-11). (C) $CD8^{+}$ $IFN\text{-}\gamma^{+}$ responses (n= 5-8). (D) $CD8^{+}TNF^{+}$ responses (n= 4-7).

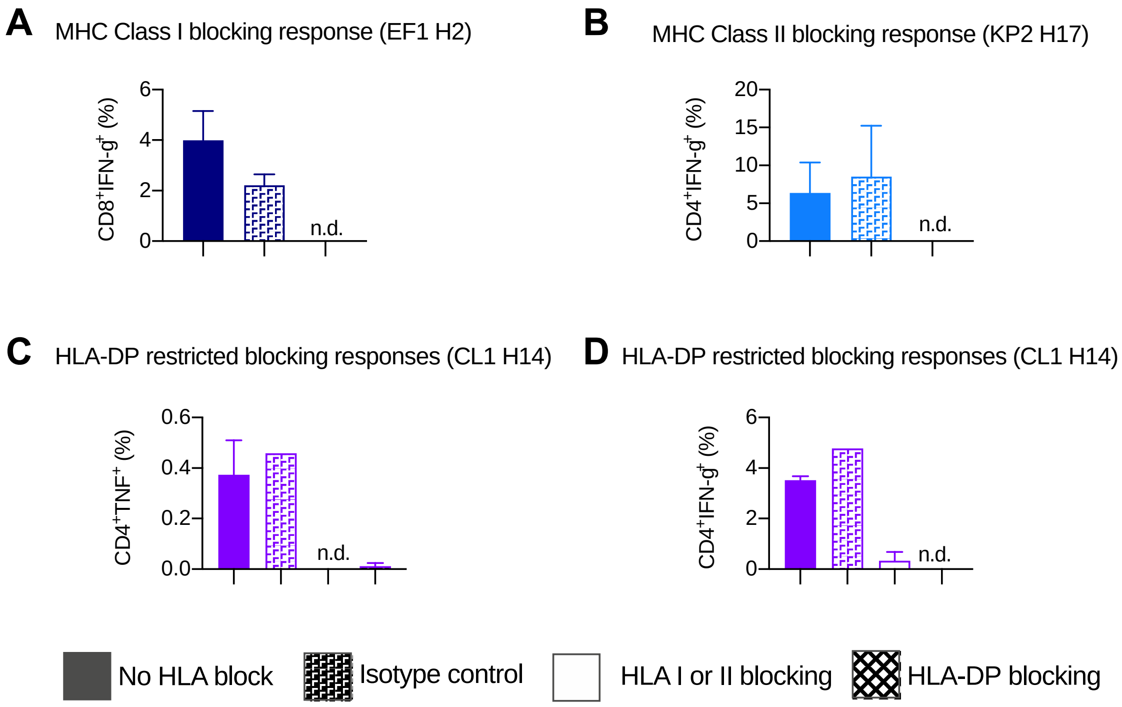

Figure S5| HLA blocking abrogates T cell responses

To verify whether the measured T cell responses were mediated by a HLA-dependent mechanism, we used antibodies to block the HLA-specific responses. Blocking the MHC class I or II almost completely abrogated T cell responses for CD8+ T cells by MHC class I blocking and in CD4+ T cells by MHC II blocking. This indicates that the peptide homologues added were being presented by MHC and causing the observed effector responses. (A) MHCI blocking $CD8^+$ IFN-γ^+ EF1 cross-reactive responses from donor H2. (B) MHCII blocking $CD4^+$ IFN-γ^+ KP2 cross-reactive responses from donor H17. (C-D) MHCII blocking $CD4^+$ TNF^+ and $CD4^+$ IFN-γ^+ CL1 cross-reactive responses from donor H14. Including pan-MHCII blocking (second last column) and specific HLA-DP blocking (last column), the HLA allele predicted to bind CL1 peptide (HLA-DR and HLA-DQ alleles from this individual were not predicted to bind CL1 peptide).

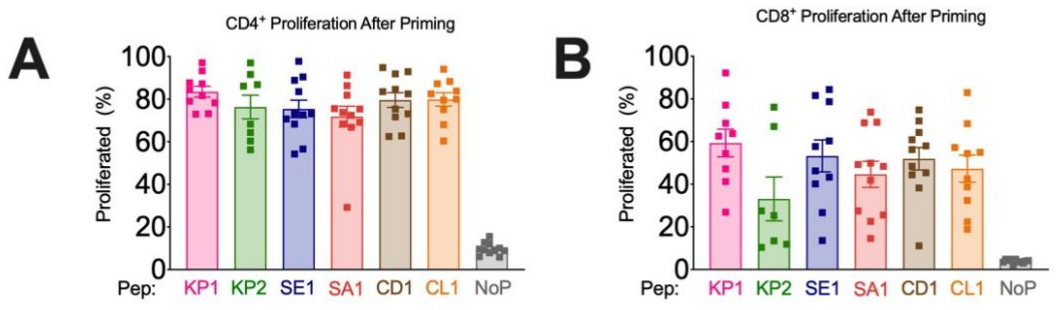

Figure S6| Priming with bacterial peptide induces T cell proliferation

Initial priming with bacterial peptide induced T cell proliferation by Cell Trace proliferation assay. (A) Percent of CD4+ T cells that underwent proliferation in response to bacterial peptides (n=8-11). (B) Percent of CD8+ T cells that underwent proliferation in response to bacterial peptides (n=7-11). NoP – no peptide negative control.

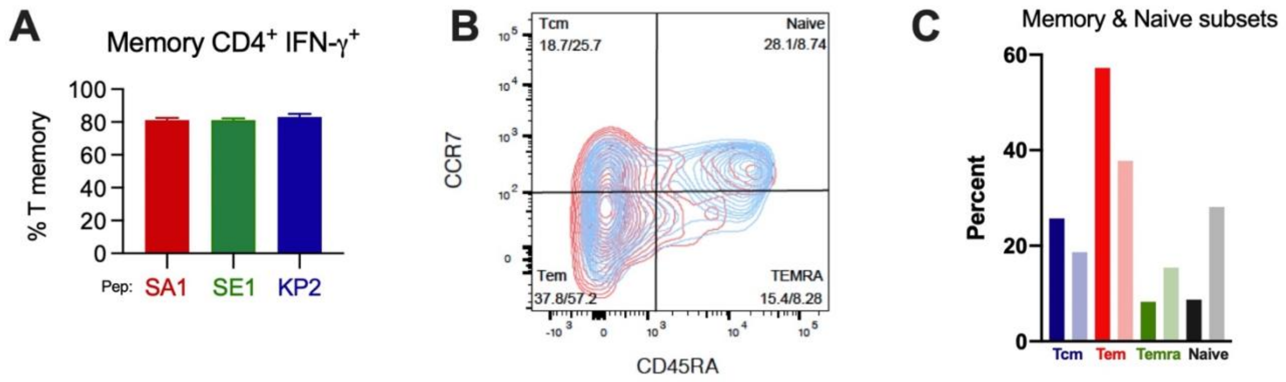

Figure S7| Post-SARS-CoV-2 cross-reactive T cells arise predominantly from memory T cells.

T cell responses against pathogenic bacterial peptides SA1, SE1 and KP2 are predominantly from a T memory cell phenotype, suggesting they arise from prior exposure to the antigen or from a mimic peptide such as from SARS-CoV-2 vaccination. (A) Percent of memory T cells present in the $CD4^+$ IFN-γ^+ population of T cells stimulated *ex-vivo* with bacterial peptide post-SARS-CoV-2 vaccination. Memory phenotype defined by CD45RA vs. CCR7 expression. (B) Contour plot of CD45RA vs. CCR7 expression on $CD4^+CD69^+$ (red contour) and total $CD4^+$ (blue contour) from T cells stimulated *ex-vivo* with SA1 bacterial peptide post-SARS-CoV-2 vaccination. Tcm- T central memory cells. Tem – T effector memory cells. TEMRA- T effector memory re-expressing CD45RA cells. Naïve- T naïve cells. Quadrant gate percentages $CD4^+CD69^+$ (left number) and total $CD4^+$ (right number). (C) Column graph of the percentages of T naïve and T memory subsets from the contour plot in C. Dark shade- $CD4^+CD69^+$ post-SARS-CoV-2 vaccination. Light shade- total $CD4^+$ post-SARS-CoV-2 vaccination.

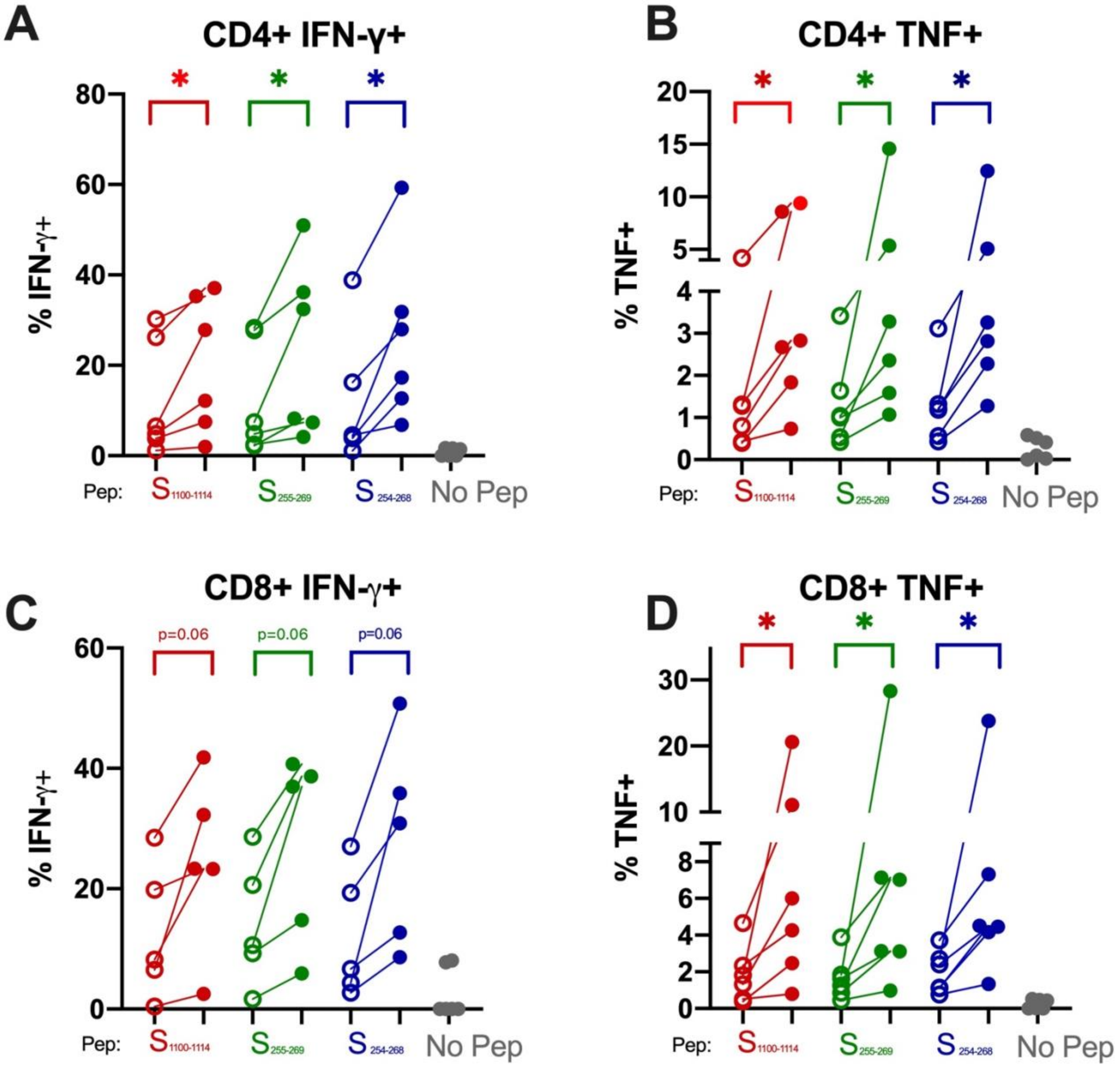

Figure S8| SARS-CoV-2-specific T cell responses before and after SARS-CoV-2 vaccination.

Direct *ex vivo* T cell responses against SARS-CoV-2 spike peptides $S_{1100-1114}$ homologous with SA1 peptide, $S_{255-269}$ homologous with SE1 peptide and $S_{254-268}$ homologous with KP2 peptide. NoP – no peptide negative control. Individual donor responses are tracked before and after SARS-CoV-2 vaccination. (A) $CD4^+$ IFN-γ^+ responses (n= 6). (B) $CD4^+$ TNF^+ responses (n= 6). (C) $CD8^+$ IFN-γ^+ responses (n= 5-6). (D) $CD8^+$ TNF^+ responses (n= 6).

Supplementary references:

1. Sulaiman I, Chung M, Angel L, Tsay JJ, Wu BG, Yeung ST, et al. Microbial signatures in the lower airways of mechanically ventilated COVID-19 patients associated with poor clinical outcome. Nat Microbiol. 2021;6(10):1245-58.

2. Bengoechea JA, Sa Pessoa J. Klebsiella pneumoniae infection biology: living to counteract host defences. FEMS Microbiology Reviews. 2019;43(2):123-44.

3. Murakami T, Hatano S, Yamada H, Iwakura Y, Yoshikai Y. Two types of interleukin 17A–producing γδ T cells in protection against pulmonary infection with Klebsiella pneumoniae. The Journal of Infectious Diseases. 2016;214(11):1752-61.

4. Passet V, Brisse S. Description of Klebsiella grimontii sp. nov. International Journal of Systematic and Evolutionary Microbiology. 2018;68(1):377-81.

5. Percival SL, Williams DW. Chapter Six - Escherichia coli. In: Percival SL, Yates MV, Williams DW, Chalmers RM, Gray NF, editors. Microbiology of Waterborne Diseases (Second Edition). London: Academic Press; 2014. p. 89-117.

6. McArthur MA, Chen WH, Magder L, Levine MM, Sztein MB. Impact of CD4+ T Cell responses on clinical outcome following oral administration of wild-type enterotoxigenic Escherichia coli in humans. PLOS Neglected Tropical Diseases. 2017;11(1):e0005291.

7. Shen H, Chen H, Ou Y, Huang T, Chen S, Zhou L, et al. Prevalence, serotypes, and antimicrobial resistance of Salmonella isolates from patients with diarrhea in Shenzhen, China. BMC Microbiology. 2020;20(1):197.

8. Ravindran R, McSorley SJ. Tracking the dynamics of T-cell activation in response to Salmonella infection. Immunology. 2005;114(4):450-8.

9. Agudelo Higuita NI, Huycke MM. Enterococcal disease, epidemiology, and implications for treatment. In: Gilmore MS, Clewell DB, Ike Y, Shankar N, editors. Enterococci: From commensals to leading causes of drug resistant infection. Boston: Massachusetts Eye and Ear Infirmary; 2014.

10. Kao PHN, Kline KA. Dr. Jekyll and Mr. Hide: How Enterococcus faecalis subverts the host immune response to cause infection. Journal of Molecular Biology. 2019;431(16):2932-45.

11. Tong SYC, Davis JS, Eichenberger E, Holland TL, Fowler VG, Jr. Staphylococcus aureus infections: epidemiology, pathophysiology, clinical manifestations, and management. Clin Microbiol Rev. 2015;28(3):603-61.

12. Bröker BM, Mrochen D, Péton V. The T cell response to Staphylococcus aureus. Pathogens. 2016;5(1):31.

13. Liu L-H, Wang N-Y, Wu AY-J, Lin C-C, Lee C-M, Liu C-P. Citrobacter freundii bacteremia: Risk factors of mortality and prevalence of resistance genes. Journal of Microbiology, Immunology and Infection. 2018;51(4):565-72.

14. Johnston PF, Gerding DN, Knight KL. Protection from Clostridium difficile infection in CD4 T Cell- and polymeric immunoglobulin receptor-deficient mice. Infect Immun. 2014;82(2):522-31.

15. Yacyshyn MB, Reddy TN, Plageman LR, Wu J, Hollar AR, Yacyshyn BR. Clostridium difficile recurrence is characterized by pro-inflammatory peripheral blood mononuclear cell (PBMC) phenotype. Journal of Medical Microbiology. 2014;63(10):1260-73.

16. Lagneaux AS, Hénard S, Diancourt L, Stein E, Perez P, Mathieu P, et al. Clostridium haemolyticum Infection: A Cause of Hemolytic Anemia in a Patient with Bone Marrow Necrosis. Microorganisms. 2021;9(8):1568.

17. Aronoff DM, Kazanjian PH. Historical and contemporary features of infections due to Clostridium novyi. Anaerobe. 2018;50:80-4.
